# Large Energy Capacitive High-Entropy Lead-Free Ferroelectrics

**DOI:** 10.1007/s40820-023-01036-2

**Published:** 2023-03-10

**Authors:** Liang Chen, Huifen Yu, Jie Wu, Shiqing Deng, Hui Liu, Lifeng Zhu, He Qi, Jun Chen

**Affiliations:** 1 Beijing Advanced Innovation Center for Materials Genome Engineering, Department of Physical Chemistry, University of Science and Technology Beijing, Beijing, 100083 People’s Republic of China; 2 School of Mathematics and Physics, University of Science and Technology Beijing, Beijing, 100083 People’s Republic of China; 3 School of Materials Science and Engineering, University of Science and Technology Beijing, Beijing, 100083 People’s Republic of China

**Keywords:** High-entropy, Energy storage, Lead-free, Relaxor ferroelectrics, Capacitors

## Abstract

Advanced lead-free energy storage ceramics play an indispensable role in next-generation pulse power capacitors market. Here, an ultrahigh energy storage density of ~ 13.8 J cm^−3^ and a large efficiency of ~ 82.4% are achieved in high-entropy lead-free relaxor ferroelectrics by increasing configuration entropy, named high-entropy strategy, realizing nearly ten times growth of energy storage density compared with low-entropy material. Evolution of energy storage performance and domain structure with increasing configuration entropy is systematically revealed for the first time. The achievement of excellent energy storage properties should be attributed to the enhanced random field, decreased nanodomain size, strong multiple local distortions, and improved breakdown field. Furthermore, the excellent frequency and fatigue stability as well as charge/discharge properties with superior thermal stability are also realized. The significantly enhanced comprehensive energy storage performance by increasing configuration entropy demonstrates that high entropy is an effective but convenient strategy to design new high-performance dielectrics, promoting the development of advanced capacitors .

**Supplementary Information:**

The online version contains supplementary material available at 10.1007/s40820-023-01036-2.

## Highlights

- Ultrahigh energy storage density of ~ 13.8 J cm^−3^ and large efficiency of ~ 82.4% are achieved in high-entropy lead-free relaxor ferroelectrics via high-entropy strategy, realizing nearly ten times growth.
- Outstanding energy storage properties are attributed to the enhanced random field and breakdown field, decreased nanodomain sizes, strong multiple local distortions coexisting in-phase and anti-phase oxygen octahedron tilts.
- Evolution of energy storage performance and domain structure with the increase in configuration entropy is systematically revealed for the first time.

**Supplementary Information:**

The online version contains supplementary material available at 10.1007/s40820-023-01036-2.

## Introduction

Pulse power capacitors are intensively used in microwave communications, hybrid electrical vehicles, medical devices, and other electronic power systems [1–4]. Lead-free dielectric ceramics, the core components of capacitors, are becoming high-profile energy storage materials owing to their distinctive features of high power density (*P*_D_), ultrafast charge/discharge rate (*t*_0.9_), and excellent operation stability [2, 5]. However, low recoverable energy density (*W*_rec_) and poor energy efficiency (*η*) largely hinder their further development toward miniaturization, lightweight and integration, meeting the demanding and extensive demands of the capacitor market. Therefore, it is essential to improve the energy storage performance of dielectric ceramics, realizing a breakthrough in performance and application [6, 7].

It is recognized that breakdown electric field (*E*_b_) and *η* are two key factors affecting *W*_rec_. The strategies of bandgap engineering and microstructure optimization are often utilized to enhance *E*_b_. For example, selecting a matrix with high band gap (*E*_g_) or introducing high-*E*_g_ solutions can essentially solve the problem of low *E*_b_ and improve the intrinsic breakdown strength [8–10], benefiting from the suppression of carrier transition under external electric field. Grain size engineering and sintering aids are regarded as frequently used paths to inhibit grain growth or reduce grain size, achieving the purpose of enhancing electromechanical breakdown strength based on compact microstructure with few pores [3, 9–12, 12]. Furthermore, heat can be continuously generated within the dielectrics because of the conductive leakage current and dielectric loss (tan*δ*), degrading the thermal breakdown resistance [3, 13], which is closely related to energy storage efficiency. Low *η* is generally obtained as a result of insufficient refinement of nanodomain, considerable antiferroelectric–ferroelectric phase transition, large tan*δ*, poor sintering characteristic, etc. To enhance *η*, some strategies, such as domain/nanodomain engineering [4, 14–16], defect engineering [17], and stabilizing antiferroelectric phase [18–21], have been proposed to enhance the random field and break the long-range ferroelectric order.

Unfortunately, different parameters or strategies could restrict each other, such as the contradiction between high *E*_b_ and maximum polarization (*P*_max_), and the significantly reduced *P*_max_ caused by the nanoscale domain refinement or ferroelectric relaxation process. Each strategy mentioned above is very difficult to achieve notable comprehensive improvement of energy storage performance alone, which typically requires perfect collaboration between multiple strategies. Coincidentally, high-entropy design concept can efficiently realize multidirectional regulation including *E*_b_, polarization, *η*, and polarization saturation behavior from multiple perspectives such as composition, microstructure, and local structure, showing excellent adjustability, diversity, and practicability [3, 4, 22]. It can be speculated that breakthrough progress in ultrahigh *W*_rec_ with excellent comprehensive performance can be realized in high-entropy ferroelectrics. However, the evolution of energy storage performance and domain structure with the increase in configuration entropy (Δ*S*_config_) has not been systematically revealed, which is very significant for designing and developing new ultrahigh-performance energy storage materials and devices.

**Fig. 1:**
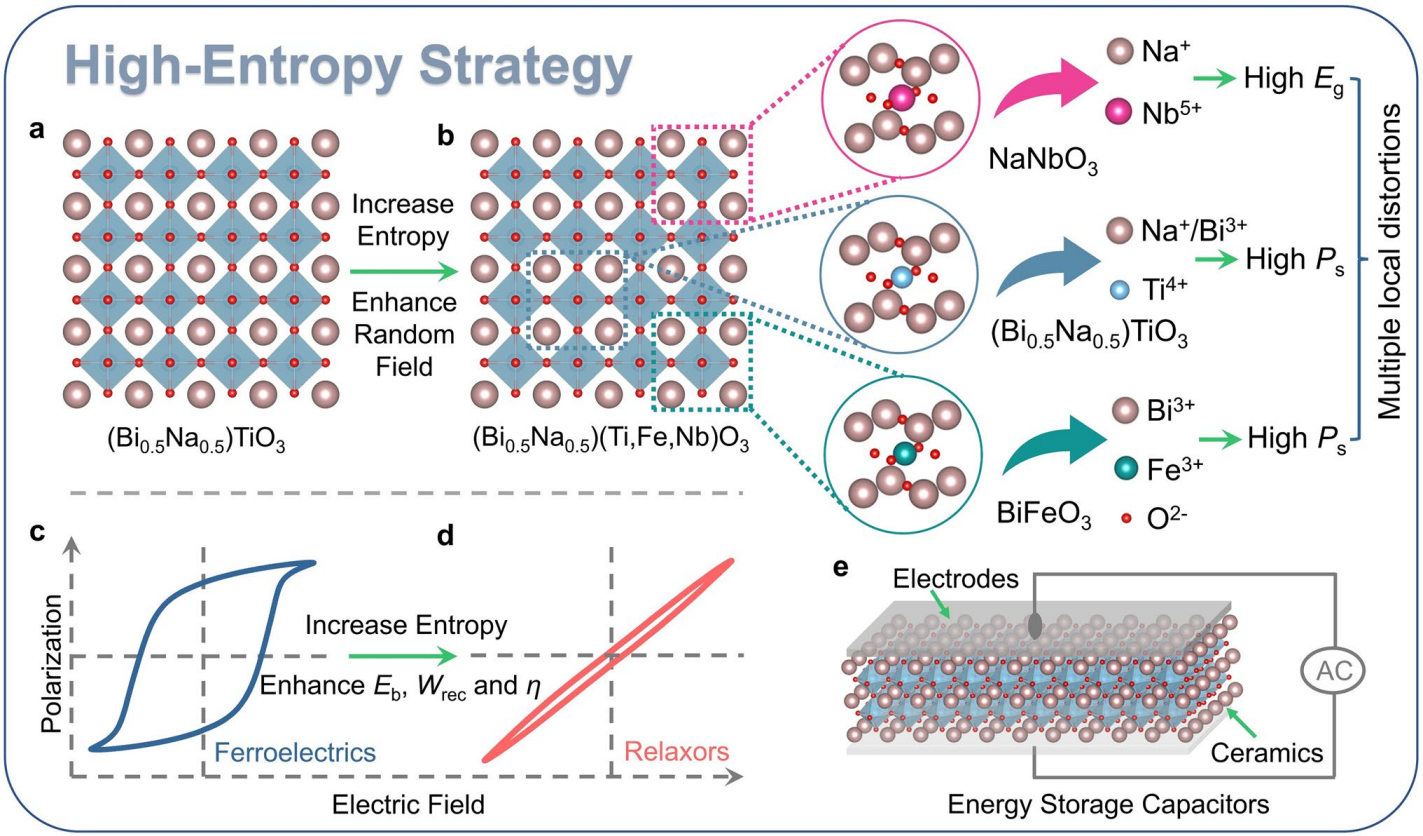
Schematic diagram of enhanced energy storage performance via high-entropy strategy. (**a**) Schematic diagram of BNT. (**b**) Schematic diagram of BNTFN-*x* and high-entropy strategy. *P*-*E* loops of (**c**) BNT and (**d**) BNTFN-*x*. (**e**) Schematic diagram of energy storage capacitors

In this work, as shown in Fig. 1, high-entropy strategy is designed and high-spontaneous polarization (*P*_s_) (Bi_0.5_Na_0.5_)TiO_3_ (BNT) is chosen as the end composition. Considering the high-*E*_g_ characteristic of NaNbO_3_ (NN) and high-*P*_s_ characteristic of BiFeO_3_ (BF), without changing the *A*-site ion configurations, two heterovalent ions Fe^3+^ and Nb^5+^ are gradually introduced into *B*-site in perovskite to increase configuration entropy, enhancing the random electric and stress fields. Furthermore, considering their own oxygen octahedron distortion features, multiple local distortions coexisting in-phase and anti-phase oxygen octahedron tilts can be formed to further enhance the random field and significantly delay polarization saturation. The elaborately designed compositions would combine the above advantages to enhance *E*_b_, improve *W*_rec_, and increase *η*, realizing the transformation of ferroelectrics to relaxor ferroelectrics. As a result, an ultrahigh *W*_rec_ of ~ 13.8 J cm^−3^ and a large *η* of ~ 82.4% are achieved in high-entropy lead-free relaxor ferroelectrics (BNTFN-1/3). Compared with the low-entropy BNTFN-0, based on the substantial improvement of *η*, the *W*_rec_ of BNTFN-1/3 has achieved nearly 10 times growth, reaching a comprehensive and huge improvement in energy storage performance. The excellent energy storage performance is mainly attributed to the decreased nanodomain size and enhanced random field caused by increasing entropy. This work systematically reveals the evolution of energy storage performance and domain structure with the increase in configuration entropy, demonstrating that high entropy is an effective but convenient strategy to design new high-performance dielectrics.

## Experimental Section

### Sample Preparation

(Bi_0.5_Na_0.5_)(Ti_1-2*x*_Fe_*x*_Nb_*x*_)O_3_ (abbreviated as BNTFN-*x*, *x* = 0, 0.1, 0.2, 0.3, 1/3) ceramics were fabricated by a conventional solid-state reaction method with the Δ*S*_config_ of 0.69*R*, 1.33*R*, 1.64*R*, 1.78*R*, and 1.79*R*, respectively, which can be calculated using the following formula in perovskite [23, 24]:

1 $$\Delta S_{{{\text{config}}}} = - R\left[ {\left( {\mathop \sum \limits_{a = 1}^{n} x_{a} lnx_{a} } \right)_{A - site} + \left( {\mathop \sum \limits_{b = 1}^{n} x_{b} lnx_{b} } \right)_{{B - {\text{site}}}} + 3\left( {\mathop \sum \limits_{c = 1}^{n} x_{c} lnx_{c} } \right)_{{O - {\text{site}}}} } \right]$$

where *x*_a_, *x*_b,_ and *x*_c_ are the mole fraction of the ions present in the A-site, B-site, and O-site, respectively. When Δ*S*_config_ < 0.69*R*, it is a low-entropy material. It belongs to medium-entropy material when 0.69R ≤ Δ*S*_config_ < 1.61*R*. When Δ*S*_config_ ≥ 1.61*R*, it is called high-entropy material. High-purity Bi_2_O_3_ (Aladdin, 99.99%), Na_2_CO_3_ (Aladdin, 99.99%), TiO_2_ (Aladdin, 99.8%), Fe_2_O_3_ (Aladdin, 99.9%), and Nb_2_O_5_ (Aladdin, 99.9%) were used as the raw materials, and 1.0 mol% MnO_2_ (99.0%) was used as a sintering aid. The dried powders were mixed by planetary mill with yttrium stabilized zirconia balls and alcohol for 24 h. Then, the mixed powders dried and calcined at 750–800 °C for 5 h. The calcined powders were mixed with 0.5 wt% PVB binder by high-energy ball milling at 600 r min^−1^ for 15 h. After drying, the powders were pressed into pellets with diameters of 10 mm under about 300 MPa. The pellets were heated to 550 °C for 2 h to burn out PVB binder and then sintered at 1050–1250 °C for 2 h with sacrificial powders in closed crucibles. The sintered samples were polished into a thickness of ~ 50–80 μm for energy storage tests, and then two parallel surfaces were covered with silver electrode and fired at 550 °C for 20 min.

### Structure Characterizations

X-ray diffraction (XRD) tests were conducted using an X-ray diffractometer (Rigaku) with Co target (*λ* = 1.79 Å). The grain morphologies and element distribution mappings of the thermal etched samples after fine polishing were detected using a scanning electron microscopy (SEM, LEO1530, ZEISS SUPRA 55, Oberkochen, Germany), and the area of 1 × 1 µm^2^ within one grain of the samples was selected for analyzing the microscopic domain configuration using piezoresponse force microscopy (PFM, Asylum Research, USA, MFP-3D). The carefully polished sample below 40 µm was further thinned by an ion milling system (PIPS, Model 691, Gatan Inc., Pleasanton, CA, USA) with a liquid nitrogen cooled stage for transmission electron microscopy (TEM) measurement. Domain morphologies and lattice fringes were observed on a field-emission TEM (JEM-2100, JEOL, Japan) with an accelerating voltage of 200 kV.

### Electric Property Measurement

*P*-*E* loops with test frequency of 10 Hz and frequency- and cycling-dependent* P*-*E* loops were tested by a ferroelectric analyzer (aix ACCT, TF Analyzer 1000, Aachen, Germany). Temperature- and frequency-dependent dielectric and loss spectra were performed using a precision LCR meter (Keysight E4990A, Santa Clara, CA) with a heating rate of 3 °C min^−1^. The charge/discharge properties of ceramics with a thickness of ~ 80 μm were performed using a commercial charge–discharge device (CFD-001, Gogo Instruments Technology, Shanghai, China).

### Finite Element Simulation

The electric field and electric potential distribution and electric tree evolution were simulated by finite element methods with 2D models using COMSOL software. The simulated model is based on the SEM diagrams, and the selected size is 16 × 24 μm^2^. The detailed calculation and simulation process are described in Supplementary Information.

### Weibull Distribution

Weibull experiments of the samples can be calculated by the following equations:

2 $$P_{i} = 1/\left( {n + 1} \right)$$

3 $$X_{i} = \ln \left( {E_{i} } \right)$$

4 $$Y_{i} = \ln \left( {\ln \left( {1/\left( {1 - P_{i} } \right)} \right)} \right)$$

where *E*_i_ is the specific breakdown strength of each ceramic, *i* presents the ordinal number of the sample, and *n* is the total amount of ceramics (*n* = 10 in this work). The intersection of the fitted line and *Y*_i_ = 0 is the theoretical *E*_b_ value.

## Results and Discussion

### Energy Storage Performance versus Configuration Entropy

The transformation from rhombohedral (R)-phase ferroelectric to pseudo-cubic phase relaxors can be clearly observed when Fe^3+^ and Nb^5+^ are gradually introduced to BNT, according to the results of XRD patterns and temperature-dependent dielectric spectra (Figs. S1 and S2). In addition, the diffraction peaks gradually shift to lower angle, demonstrating that Fe^3+^ and Nb^5+^ successfully replace Ti^4+^ at B-site and new solid solutions are formed through high-entropy design, which is attributed to the higher ionic radii of Fe^3+^ and Nb^5+^.

Energy storage performance is closely related to structure. As shown in Fig. S3, BNTFN-0 ceramic exhibits a normal ferroelectric bipolar loop with high remnant polarization (*P*_r_), large hysteresis, and obvious polarization saturation phenomenon. The *W*_rec_ and *η* of BNTFN-0 ceramic under 10 kV mm^−1^ are only 0.39 J cm^−3^ and 8.4%, respectively. With the introduction of Fe^3+^ and Nb^5+^ or the increase in Δ*S*_config_, the ferroelectric hysteresis loop gradually changes from a normal ferroelectric to a relaxor ferroelectric characteristic accompanied by the significant decrease in *P*_r_ and *P*_max_ as well as the increase in *η*.

**Fig. 2:**
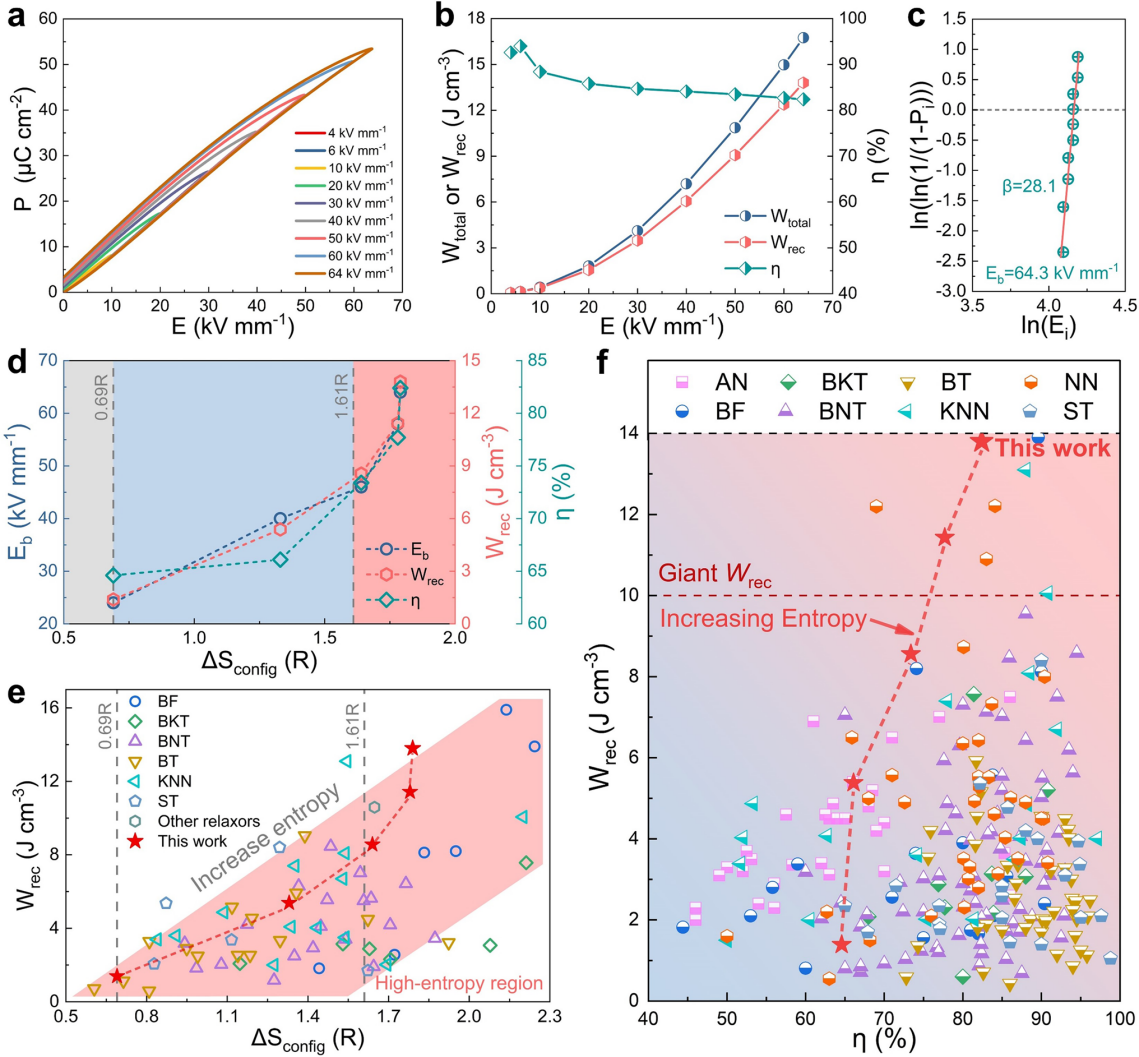
Ultrahigh energy storage performance by increasing configuration entropy. (**a**) *P*-*E* loops, and (**b**) *W*_rec_ and *η* of BNTFN-1/3 ceramics under various electric fields. (**c**) Weibull distribution of *E*_b_ for BNTFN-1/3 ceramics. (**d**) *E*_b_, *W*_rec_, and *η* as a function of Δ*S*_config_ for BNTFN-1/3 ceramics. (**e**) A comparison of *W*_rec_ and Δ*S*_config_ between the studied samples in this work and other reported lead-free relaxor ferroelectric ceramics. (**f**) A comparison of *W*_rec_ and *η* between the studied samples in this work and other reported lead-free ceramics (AN: AgNbO_3_; BKT: Bi_0.5_K_0.5_TiO_3_; BT: BaTiO_3_; KNN: (K,Na)NbO_3_; ST: SrTiO_3_)

To explore the energy storage potential of the studied samples, unipolar *P*-*E* loops are tested from low electric field to breakdown field, as recorded in Figs. 2a–b and S4. As the increase in Fe^3+^ and Nb^5+^ content, the polarization saturation behaviors are significantly delayed and the hysteresis is significantly reduced, indicating the enhanced relaxation behavior and random field, which demonstrates the lack of macro-domain and the breaking of long-range ferroelectric order. In addition, both of *W*_total_ and *W*_rec_ in high-entropy BNTFN-0.2, BNTFN-0.3, and BNTFN-1/3 samples show a nearly parabolic growth trend with the applied electric field till *E*_b_. As shown in Fig. 2d, low-entropy BNTFN-0 ceramic shows the lowest *W*_rec_ ~ 1.39 J cm^−3^ and *η* ~ 64.6% than medium-entropy (BNTFN-0.1) and high-entropy samples (BNTFN-*x*, *x* = 0.2, 0.3, 1/3). It should be noted that the larger *η* under unipolar *P*-*E* loops than that of bipolar loops could be caused by the finite reversible domain switching [4]. When low-entropy material evolves into medium-entropy material (from 0.69*R* to 1.33*R*), the *W*_rec_ of BNTFN-0.1 sample (~ 5.38 J cm^−3^) is about 4 times that of BNTFN-0. When Δ*S*_config_ continues to increase to the high-entropy level, the ultrahigh energy storage performance can be realized in BNTFN-0.2 (Δ*S*_config_ ~ 1.64*R*, *W*_rec_ ~ 8.56 J cm^−3^) and BNTFN-0.3 (Δ*S*_config_ ~ 1.78*R*, *W*_rec_ ~ 11.43 J cm^−3^) ceramics. Encouragingly, the highest *W*_rec_ of ~ 13.8 J cm^−3^ and maximum *η* of ~ 82.4% are achieved in BNTFN-1/3 relaxor ferroelectrics with the highest configuration entropy (Δ*S*_config_ ~ 1.79*R*). Compared with the low-entropy BNTFN-0, based on the substantial improvement of *η*, the *W*_rec_ of BNTFN-1/3 has achieved nearly 10 times growth, reaching a comprehensive and huge improvement in energy storage performance. Furthermore, the enhanced energy storage performance is inseparable from the largely improved *E*_b_ from 24 to 64 kV mm^−1^ as the increase in Δ*S*_config_. The *E*_b_ of ~ 62 kV mm^−1^ in BNTFN-0.3 ceramic is similar to that of BNTFN-1/3 due to the approximate Δ*S*_config_, indirectly showing the high reliability of the obtained ultrahigh *E*_b_ and energy storage performance in BNTFN-1/3 ceramics. As counted in Fig. 2c, the linear relationships with the Weibull modulus (*β*) value are 28.1 and the theoretical *E*_b_ value of BNTFN-1/3 sample is 64.3 kV mm^−1^, which is very close and slightly higher than the experimental *E*_b_ value, implying again the validity of the Weibull distribution and energy storage properties [4, 25].

To understand the evolution path of the studied samples in the whole lead-free energy storage system with the increase in Δ*S*_config_, the comparisons of *W*_rec_ and Δ*S*_config_ as well as *W*_rec_ and *η* are systematically conducted. As summarized in Fig. 2e and Table S1, the reported *W*_rec_ of relaxor ferroelectrics shows an increasing trend with the increase in Δ*S*_config_ on the whole, and the ultrahigh *W*_rec_ is clearly found near or in the high-entropy region. Furthermore, the high-entropy samples in this work have lower element types (*n* = 6) than other reported ultrahigh-performance systems (*n* ≥ 10, *W*_rec_ ≥ 10 J cm^−3^) [3, 26, 27]. If the types of elements continue to increase to further improve Δ*S*_config_, it is speculated that their *W*_rec_ and *η* will significantly increase. However, at present, the reported ultrahigh *W*_rec_ for (relaxor) antiferroelectrics tends to be obtained near the low-entropy or medium-entropy region (Fig. S5), which may be related to the phase transition regulation between antiferroelectric and ferroelectric at the expense of sacrificing efficiency. As a result, high-entropy antiferroelectric materials are rarely reported, but they still need to be further designed or attempted. As shown in Fig. 2f and Table S1, it is clearly shown that the comprehensive energy storage properties (*W*_rec_ and *η*) of the studied samples are gradually increase with the increase in configuration entropy, breaking through the energy storage level of 10 J cm^−3^ in the high-entropy stage of 1.78*R* and 1.79*R*. Entropy measures the degree of chaos in the system. The increase in entropy in energy storage ceramics means the enhancement of random field, which is conducive to breaking the long-range ferroelectric order and forming nanoscale domains or PNRs, leading to a significantly improved *η*. The outstanding energy storage performance (*W*_rec_ of ~ 13.8 J cm^−3^ and *η* of ~ 82.4%) realized in BNTFN-1/3 high-entropy ceramics shows great superiority compared with other lead-free systems. The results show that the high-entropy strategy is a very effective design to enhance the comprehensive energy storage performance of materials.

### Evolution of Domain Structure versus Configuration Entropy

**Fig. 3:**
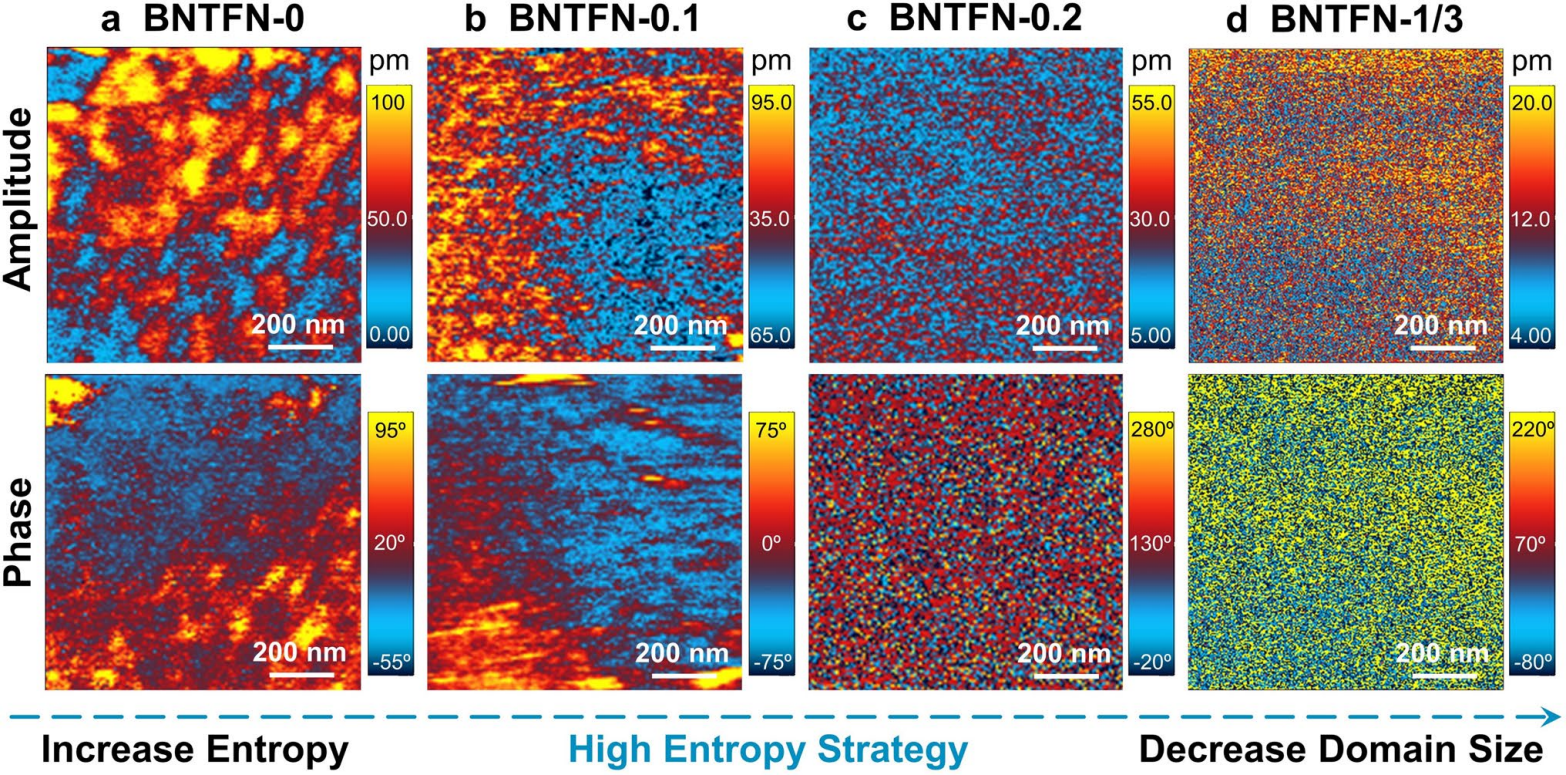
Transformation of domain morphology by increasing configuration entropy. Out-of-plane PFM amplitude and phase images of (**a**) BNTFN-0, (**b**) BNTFN-0.1, (**c**) BNTFN-0.2, and (**d**) BNTFN-1/3 ceramics

The control of domain structure plays an important role in energy storage performance. It is recognized that long-range ferroelectric order is usually accompanied by large-size domains. To explore the evolution of domain structure with the increase in entropy, the out-of-plane PFM is conducted within one grain in each sample after polished and thermally etched (Fig. S6). As shown in Fig. 3a, it is clearly shown that BNTFN-0 sample exhibits large-size nanodomains with about 100–200 nm, which is consistent with the previously reported PFM and TEM results [28, 29]. Large hysteresis in BNTFN-0 ceramic is mainly ascribed to large ferroelectric nanodomains, leading to strong domain switching under electric fields. After 0.1 mol Fe^3+^ and Nb^5+^ are doped into BNT to enhance the random field or Δ*S*_config_, dozens of nanometer domains can be obviously identified in medium-entropy BNTFN-0.1 material (Fig. 3b). When the entropy increases to the high-entropy level (Δ*S*_config_ ≥ 1.61*R*), the domain size continues to decrease so that it cannot be effectively identified in accordance with the chaotic amplitude and phase response (Fig. 3c-d), especially in BNTFN-1/3 sample, which is limited by the resolution of PFM. According to the previous reports, there may be ultrasmall-size PNRs with several nanometers in the high-entropy samples that domain morphology cannot be well discriminated by PFM [3, 4, 9, 30]. The local random field enhanced by high entropy greatly destroys the long-range ordered ferroelectric state, driving the evolution of domain structure from large-scale domains to small-scale domains and even PNRs, which results in a large improvement of *W*_rec_ and *η* owing to the enhanced activity and response speed under external electric field [3, 4, 31]. Furthermore, on the basis of PFM amplitude results, the maximum amplitude shows a downward trend on the whole, indicating reduced ferroelectric activity and enhanced relaxation behavior by increasing entropy.

### Local Structure and Multiple Local Distortions

**Fig. 4:**
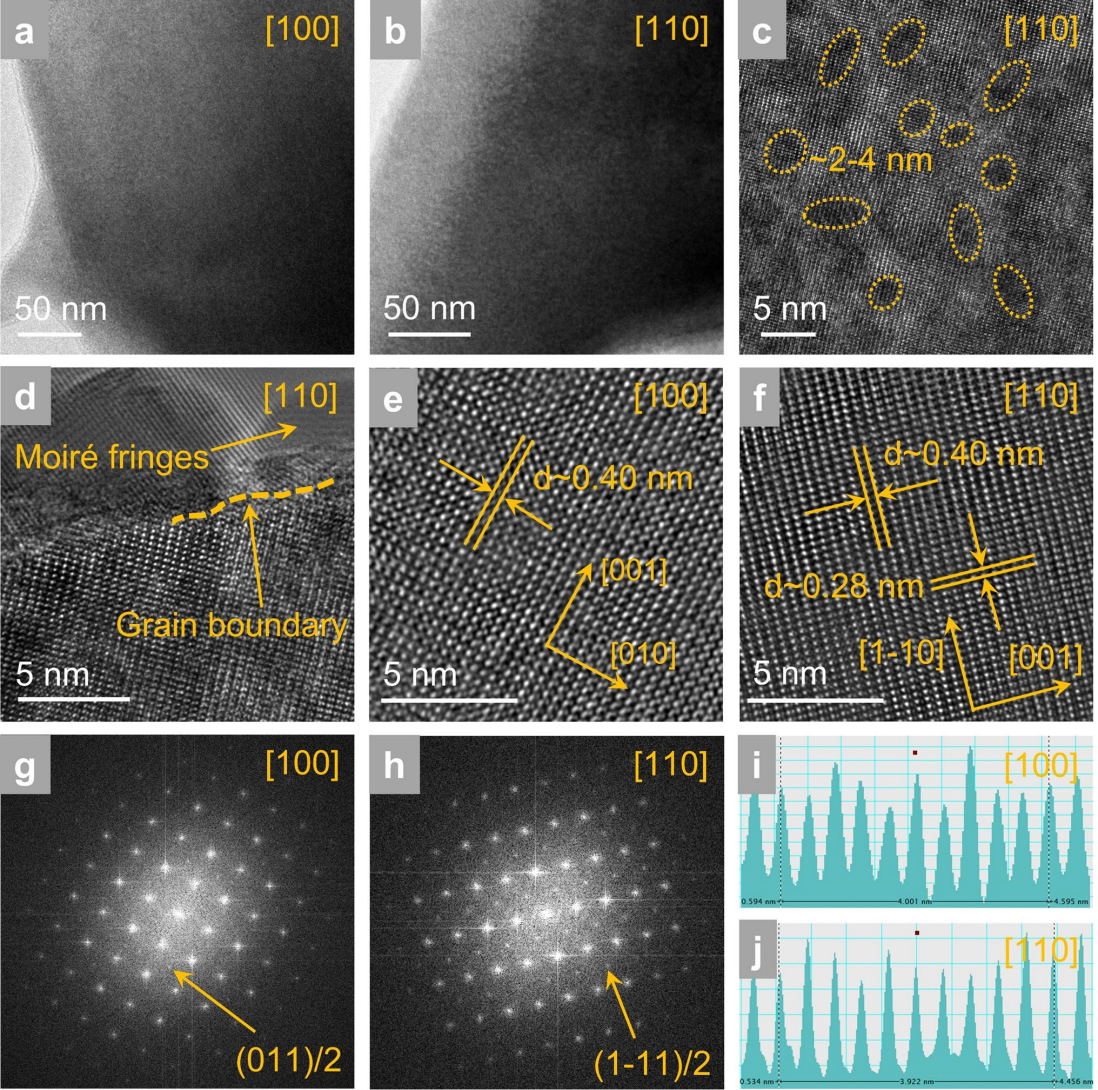
Local structure for BNTFN-1/3 ceramics. TEM images of domain morphology along (**a**) [100]_c_ and (**b**) [110]_c_. (**c**) HR-TEM pattern of domain morphology along [110]_c_. (**d**) HR-TEM pattern of Moiré fringes and grain boundary along [110]_c_. Lattice fringes along (**e**) [100]_c_ and (**f**) [110]_c_. SAED patterns after fast Fourier transform along (**g**) [100]_c_ and (**h**) [110]_c_. Atomic column intensity along (**i**) [100]_c_ and (**j**) [110]_c_

To deeply explore the structural nature for achieving ultrahigh energy storage properties, high-resolution TEM (HR-TEM) is performed for high-entropy BNTFN-1/3 ceramic. Speckle like domain morphology can be observed along [100]_c_ and [110]_c_ in Fig. 4a–b. It is known that small-size PNRs cannot be clearly revealed due to the insufficient resolution of TEM. However, through further observation, the nearly elliptical regions with different contrast are found in HR-TEM (Fig. 4c), which could be related to the existence of polarization along different directions [9, 32]. The regions with the size of about 2–4 nm are also consistent with the previous reported results of PNRs observed by atomic-resolution scanning transmission microscopy [1, 3, 4]. Furthermore, PNRs revealing as Moiré fringe structures can be observed by HR-TEM (Fig. 4d), which originate from the interference of two overlaid lattice patterns with mismatched orientations and imply the polar lattice distortion at local scale, supporting the formation of PNRs in perovskite materials [30, 33]. On the one hand, ultrasmall PNRs realized by high-entropy strategy can make the flexible polarization reorientation process with small stress under external electric field, resulting in the enhanced polarization texture along the direction of electric field and providing the basic for large *P*_max_. On the other hand, the enhanced random field with weakly coupled PNRs in high-entropy sample would delay the formation of polarization texture during electric field loading and drive the long-range ordering ferroelectric state back to the initial macro-nonpolar state during unloading, leading to delayed polarization saturation and near-zero *P*_r_, respectively [3]. Therefore, it is speculated that ultrasmall PNRs should exist in high-entropy BNTFN-1/3 ceramic, promoting a boost in energy storage performance.

Oxygen octahedron distortions have prominent influence on material properties including energy storage performance. As shown in Fig. 4e–j, lattice fringes with very uniform intensity and clear selected area electron diffraction (SAED) after fast Fourier transform can be found in BNTFN-1/3 ceramic along [100]_c_ and [110]_c_, showing a good sample quality with perovskite structure. Interestingly, both (ooe)/2 and (ooo)/2 superlattice diffractions can be detected (o is odd and e is even) in Fig. 4g–h, which demonstrates that in-phase and anti-phase oxygen octahedral tilts can be identified in the studied sample, respectively, according to the conclusions by *Glazer* [34]. Pure BNT (BNTFN-0) exhibits room-temperature R phase with *R*3c space group, which is accompanied with Bi^3+^/Na^+^ and Ti^4+^ displaced parallel to each other along [111]_c_ directions, and only (ooo)/2 can be reflected due to the single existing anti-phase oxygen octahedron tilt with a^−^a^−^a^−^ [29, 35, 36]. BF also shows room-temperature *R*3c space group but NN exhibits room-temperature antiferroelectric P phase with a^−^b^∗^a^−^ (b^∗^  = AACC) tilting system based on *Peel* notation [36–38]. Therefore, the induced BF and NN by high-entropy strategy cause coexisting in-phase and anti-phase oxygen octahedral tilts, forming multiple local distortions rather than single distortion type. It is speculated that the existence of multiple local distortions further increases the disturbance degree of the system and enhances the random field. Combined with the strong random field caused by high-entropy design, the tilt distortions of the oxygen octahedron can cause more electric energy to be absorbed during the process of forming long-range ferroelectric order from nanodomains or PNRs under external electric fields, resulting in significant delayed polarization saturation behavior (Figs. 2a and S4a, c, e, g) [3].

### Dielectric Breakdown and Electric Tree Evolution

**Fig. 5:**
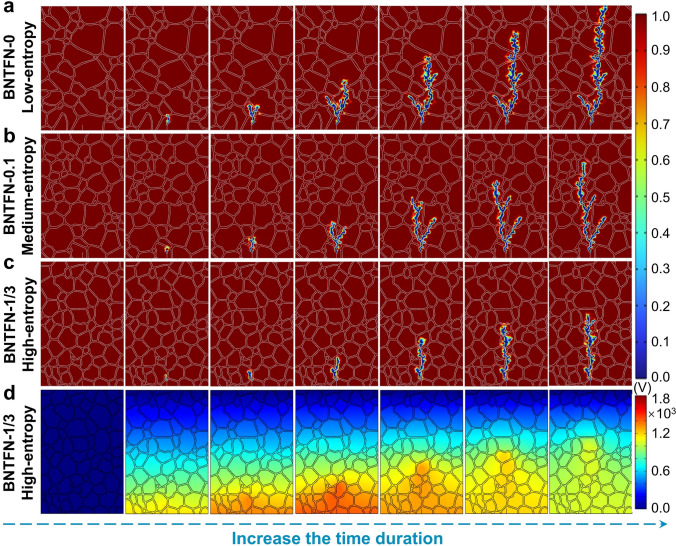
Simulation of breakdown path. Breakdown path distribution or electrical tree evolution for (**a**) BNTFN-0, (**b**) BNTFN-0.1, and (**c**) BNTFN-1/3 ceramics from low entropy to high entropy. (**d**) Electric potential distribution for BNTFN-1/3 high-entropy ceramic

Ultrahigh *E*_b_ obtained in high-entropy samples is an important aspect responsible for ultrahigh energy storage properties, which can be influenced by many factors. As shown in Fig. S7, all samples exhibit very dense grain structure without obvious pores, which is an important guarantee for high *E*_b_. Densely bonded grain boundaries can also be clearly observed in Fig. 4d by TEM. The average grain size (*G*_a_) of BNTFN-*x* (*x* = 0, 0.1, 0.2, 0.3, 1/3) samples is 3.0, 2.5, 1.4, 1.9, and 2.2 μm, respectively, showing a downward trend from low entropy to high entropy. However, *G*_a_ gradually increases when the degree of high entropy further increases, which shows that there are other key factors affecting *E*_b_. As discussed above, decreased nanodomain size can be found by increasing Δ*S*_config_ and ultrasmall PNRs can be obtained in high-entropy samples, which can lead to easier polarization reorientation under an external electric field and generate a small amount of heat, resulting in largely enhanced thermal breakdown strength [3, 4, 30]. In addition, the tan*δ* of BNTFN-1/3 sample can be effectively maintained below 0.04 from room temperature to 150 °C under different frequencies (Fig. S2f), indicating stable low-loss behaviors for improved *E*_b_. To further understand the dielectric breakdown process and the origin of ultrahigh-*E*_b_ for high-entropy material, electric field and electric potential distribution and electric tree evolution are simulated by finite element methods with 2D models in Figs. 5 and S8. The model mainly considers the intrinsic property (dielectric constant: *ε*_r_) and external structural characteristic (grain and grain boundary distribution) of materials to further simulate the most realistic results. It is recognized that strong electric field and weak electric field are concentrated in grain boundary and grain, respectively, due to low *ε*_r_ of grain boundary and high *ε*_r_ of grain. Based on this feature, grain boundary is the main barrier to the propagation of electric tree [7, 39]. With the increase in Δ*S*_config_, the red regions in grain boundaries with high electric field decrease and electric field distribution tend to be more uniform (Fig. S8a-c), avoiding the occurrence of large local electric fields and making it more difficult to break down for high-entropy materials. As shown in Fig. 5a–c, the electric tree of the studied samples gradually propagates through the grains by increasing time duration under the same electric field. The propagation speed slows down with the increase in Δ*S*_config_, showing an enhanced breakdown strength, leading to earlier breakdown for low-entropy BNTFN-0 ceramic according to the electrical tree running through the whole material. The electric potential difference near the top of electrical tree gradually reduces due to the energy dissipation with the evolution of the electrical tree (Figs. 5d and S8d-e) [7]. Furthermore, compared with low-entropy and medium-entropy samples, more uniform electric potential distribution behavior can be obviously observed in high-entropy BNTFN-1/3 sample, which is mainly attributed to the smaller grain size with more uniform distribution. Combining with the *ε*_r_ decreasing with the increase in Δ*S*_config_, the higher grain boundary density can dissipate more energy during the propagation of electric tree under external electric field, resulting in an enhanced *E*_b_ when the material evolves from low-entropy to high entropy. Consequently, ultrahigh *E*_b_ of 64 kV mm^−1^ can be realized in high-entropy BNTFN-1/3 sample, leading to a significant improvement of *W*_rec_.

### Frequency/Cycling Stability and Charge/Discharge Performance

**Fig. 6:**
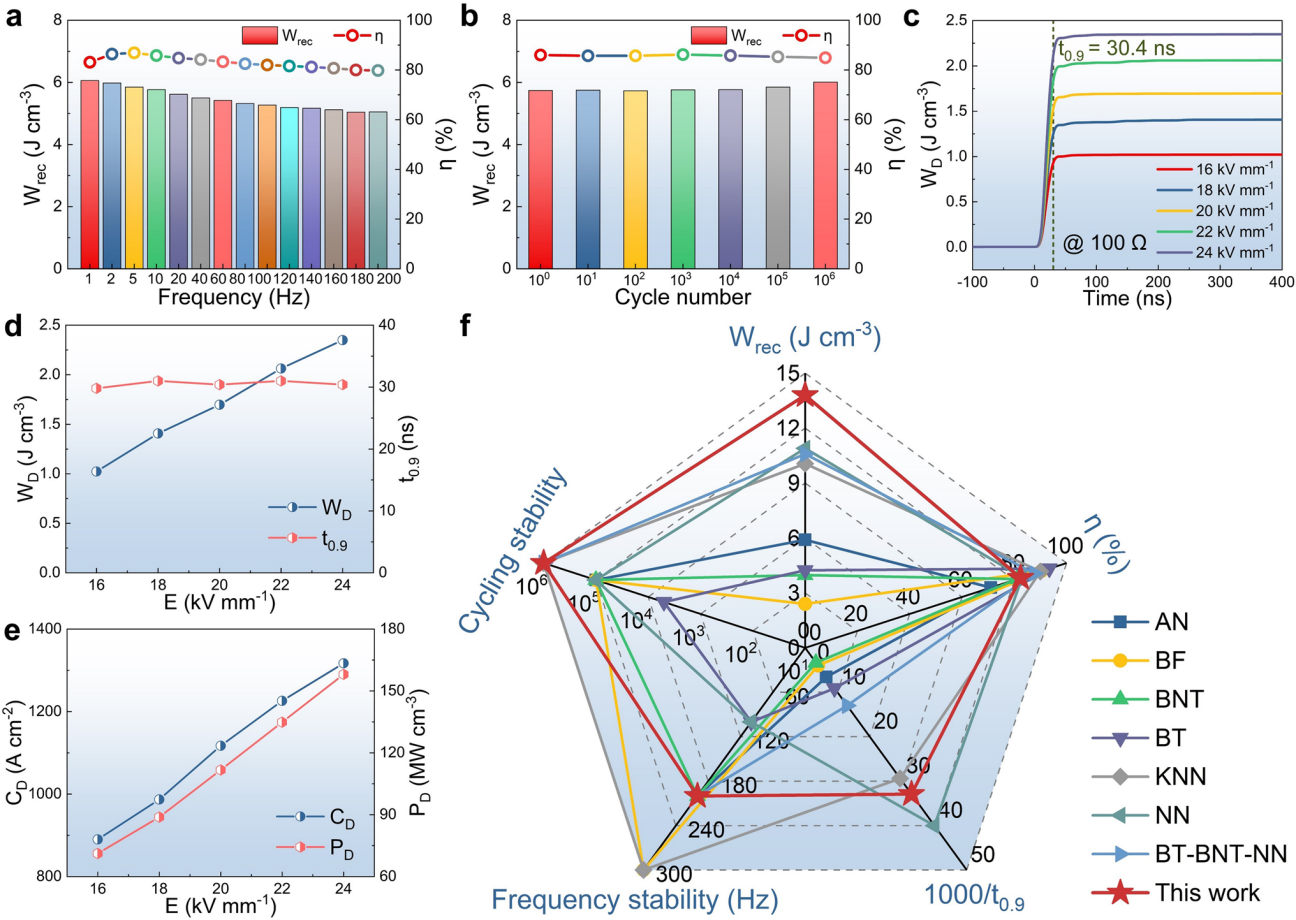
Stability and charge/discharge properties of BNTFN-1/3 energy storage ceramics. (**a**) Frequency-dependent *W*_rec_ and *η* under 40 kV mm^−1^. (**b**) *W*_rec_ and *η* as a function of the cycle number under 40 kV mm^−1^. (**c**) Calculated overdamped discharge density *W*_D_, and (**d**) *W*_D_ and *t*_0.9_ values under different electric fields (*R*_o_ = 100 Ω). (**e**) *C*_D_ and *P*_D_ values in underdamped discharge circuit under different electric fields. (**f**) Comparisons of comprehensive properties (*W*_rec_, *η*, *t*_0.9_, frequency and cycling stability) between BNTFN-1/3 ceramic and some representative energy storage ceramics with excellent comprehensive performance of different systems

Frequency and cycling stability play an important role for energy storage capacitors working at room temperature under external electric fields. Figure S9a shows the frequency-dependent *P*-*E* loops under 40 kV mm^−1^, which are slim at a very wide range from 1 to 200 Hz. As a result, ultrahigh *W*_rec_ (> 5 J cm^−3^) and large *η* (> 80%) can be well maintained (Fig. 6a), indicating the excellent frequency stability of high-entropy BNTFN-1/3 ceramics. The cycling or fatigue stability of performance is a key index to measure the service life of energy storage devices. As shown in Figs. 6b and S9b, when the electric field is cycled up to 10^6^ times, slim *P*-*E* loops exhibit nearly no change with stable *P*_max_. The cycling-insensitive energy storage performance (*W*_rec_ ~ 5.88 ± 0.14 J cm^−3^, *η* ~ 85.5 ± 0.5%) can also be obtained, showing the huge potential for practical applications.

Charge/discharge performance is measured to estimate the discharge power and rate of the high-entropy BNTFN-1/3 energy storage ceramics. For overdamped discharge process, a fixed load resistance (*R*_o_) of 100 Ω is applied at various electric fields from 16 to 24 kV mm^−1^ and temperatures from 20 to 160 °C under 20 kV mm^−1^, showing stable overdamped oscillating waveforms (Fig. S10a-b). As shown in Figs. 6c–d and S10c-d, the high discharge energy density (*W*_D_) of ~ 2.35 J cm^−3^ and ultrafast *t*_0.9_ of ~ 30.4 ns can be realized at electric field of 24 kV mm^−1^ and considerable discharge properties (*W*_D_ ≥ 1.10 J cm^−3^ and *t*_0.9_ ≤ 34.8 ns) can be effectively kept over a wide temperature range from 20 to 160 °C at 20 kV mm^−1^. At underdamped discharge circuit, regular oscillating waveforms can be clearly found in Fig. S11a. The current density (*C*_D_) and *P*_D_ increase from 890.0 to 1317.0 A cm^−2^ and from 71.2 to 158.0 MW cm^−3^, respectively, with electric field which increases from 16 to 24 kV mm^−1^ (Fig. 6e). In addition, as shown in Fig. S11b-c, stable underdamped discharge waveforms and ultrahigh performance (*C*_D_ ≥ 1355.6 A cm^−2^ and *P*_D_ ≥ 135.6 MW cm^−3^) can be well maintained under 20 kV mm^−1^ with a wide temperature range from 20 to 160 °C. A comparison of comprehensive electrical properties between BNTFN-1/3 high-entropy ceramic and other representative compositions of various lead-free systems is given in Fig. 6f [3, 4, 40–44]. It is clear that significant advantages in energy storage performance, frequency/cycling stability, and discharge performance can be realized in BNTFN-1/3 ceramic, especially that higher energy storage properties can be well maintained at high frequency and cycle number than that of other ceramics. In view of these, BNTFN-1/3 ceramic shows excellent comprehensive electrical properties, making a good prospect for pulse power capacitors.

## Conclusion

In summary, an ultrahigh *W*_rec_ of ~ 13.8 J cm^−3^ is realized in a new lead-free relaxor via high-entropy strategy, which has achieved a significant growth of nearly ten times compared with low-entropy material, accompanied by a larger *η* of ~ 82.4%. The evolution of energy storage properties and domain structure with the increase in configuration entropy has been systematically revealed in this work. The enhanced random fields, decreased nanodomain sizes, strong multiple local distortions, and improved *E*_b_ can be achieved by increasing configuration entropy, which are responsible for the great improvement of comprehensive energy storage performance with excellent frequency and fatigue stability. This work demonstrates that high entropy is an effective but convenient strategy to design new high-performance dielectrics, promoting the development of advanced pulse power capacitors.

### Supplementary Information

Below is the link to the electronic supplementary material.

Supplementary file1 (DOCX 12483 KB)
