# Supplementary material for: Large Energy Capacitive High-Entropy Lead-Free Ferroelectrics
Source: Nanomicro Lett. 2023 Mar 10;15:65. doi: 10.1007/s40820-023-01036-2 (PMC10006382; doi:10.1007/s40820-023-01036-2)
Supplement: Supplementary file 1 — Supplementary file1 (DOCX 12483 KB) [file 40820_2023_1036_MOESM1_ESM.docx]

**Supplementary Information**

**Large Energy Capacitive High-Entropy Lead-Free Ferroelectrics**

Liang Chen^1^, Huifen Yu^1,2^, Jie Wu^1,2^, Shiqing Deng^1,2^, Hui Liu^1,2^, Lifeng Zhu^3^, He Qi^1,^* and Jun Chen^1,^*

^1^ Beijing Advanced Innovation Center for Materials Genome Engineering, Department of Physical Chemistry, University of Science and Technology Beijing, Beijing 100083, China

^2^ School of Mathematics and Physics, University of Science and Technology Beijing, Beijing 100083, China

^3^ School of Materials Science and Engineering, University of Science and Technology Beijing, Beijing 100083, China

*Corresponding author. E-mail: [qiheustb@ustb.edu.cn](mailto:qiheustb@ustb.edu.cn); [junchen@ustb.edu.cn](mailto:junchen@ustb.edu.cn)

**Finite element simulation**

The electric field and electric potential distribution as well as electric tree evolution were simulated by finite element methods with 2D models using COMSOL software. The simulated model is based on the SEM diagrams and the selected size is 16×24 μm^2^. To simulate the dielectric breakdown behavior of the studied samples from low-entropy to high-entropy, a scalar field *s*(*x*, *t*) was applied to represent the breakdown state, where *s* =1 means the initial state and *s* =0 means complete breakdown state (0≤ s ≤1). Since dielectric constant (*ε*_r_) is a continuous function of *s*, the difference in *ε*_r_ is adopted to describe the breakdown state, which can be expressed as [1, 2]:

$$\varepsilon\left( s \right)=\frac{\varepsilon_{ini}}{f\left( s \right)+\psi} (1)$$

where *ε*_int_ represents the initial *ε*_r_, $f\left( s \right)=4s^{3}-3s^{4}$, and $\psi$ is 0.0001. In addition, the ferroelectric ceramics include grains and grain boundaries. The *ε*_r_ of grains is electric field-dependent following Johnson’s approximation [3], and the *ε*_r_ of grain boundary is linear. Therefore, the *ε*_r_ of grains (*ε*_g_) and grain boundaries (*ε*_gb_) under a specific electric field is described by the following equation:

$$\varepsilon_{ini}\left( E \right)=\frac{\varepsilon_{g}\left( 0 \right)}{\left( 1+kE^{2} \right)^{1/3}} (2)$$

$$\varepsilon_{ini}\left( E \right)=\varepsilon_{gb} (3)$$

where *k* is 0.0013, and *ε*_g_(0) is the zero-field dielectric constant and is taken to be 750, 700, and 500 for low-entropy BNTFN-0, medium-entropy BNTFN-0.1, and high-entropy BNTFN-1/3 samples, respectively. Generally, the ratio of *ε*_g_ to *ε*_gb_ is set as 10:1 in this work [4, 5]. The theoretical model can be constructed by the following formulas:

$$\overline{\nabla}\left[ \frac{1}{f\left( s \right)+\psi}\overline{\nabla\phi} \right]=0 (4)$$

$$\frac{\partial s}{\partial t}=-\frac{f^{'}\left( s \right)}{2\left[ f\left( s \right)+\psi\right]^{2}}\overline{\nabla\phi}\times\overline{\nabla\phi}+f^{'}\left( s \right)+\frac{1}{2}\overline{\nabla}^{2}s (5)$$

Under the correct boundary and initial conditions, Eqs. (4) and (5) can be applied to the solution of dimensionless unknown fields $\phi(x, t)$ and *s*(*x*, *t*). The simulated results are recorded in Fig. 5 and Fig. S8.


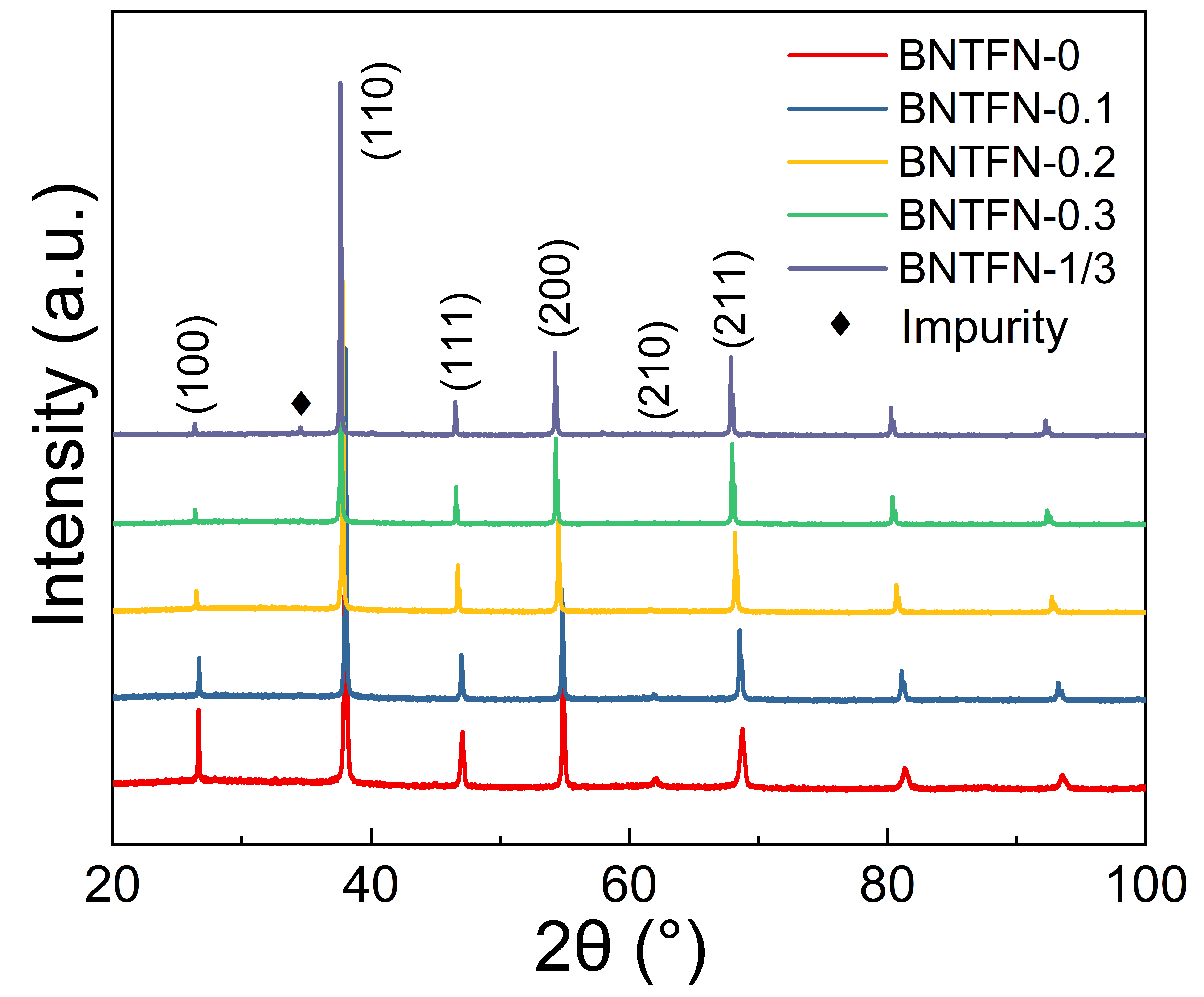


**Fig. S1** The XRD patterns for BNTFN-*x* (*x*=0, 0.1, 0.2, 0.3, 1/3) ceramics

The XRD patterns of the studied samples indicate that BNTFN-0, BNTFN-0.1, BNTFN-0.2, and BNTFN-0.3 are pure perovskite structure. A small amount of impurity can be found in BNTFN-1/3 ceramics with pseudo-cubic phase.


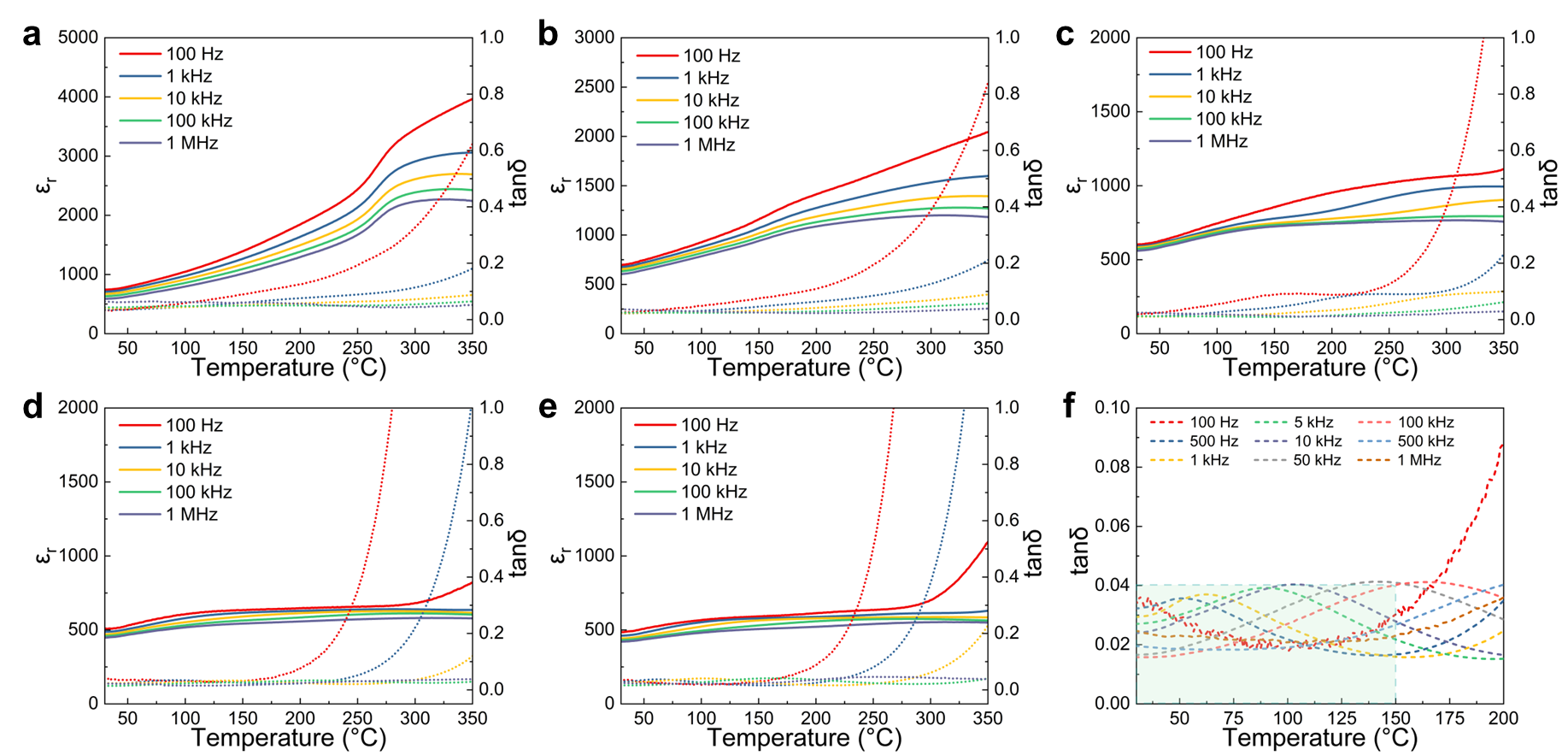


**Fig. S2** Temperature-dependent *ε*_r_ and tan*δ* for **a** BNTFN-0 ceramics, **b** BNTFN-0.1 ceramics, **c** BNTFN-0.2 ceramics, **d** BNTFN-0.3 ceramics, and **e** BNTFN-1/3 ceramics. **f** Temperature-dependent tan*δ* for BNTFN-1/3 ceramics from 100 Hz to 1 MHz

As shown in Fig. S2a, BNTFN-0 ceramic shows normal ferroelectric features. With the introduction of Fe^3+^ and Nb^5+^, enhanced diffuse phase transition behavior with flatten temperature-dependent *ε*_r_ spectra and decreased room-temperature *ε*_r_ are clearly found, implying the improved dielectric relaxation characteristic. Moreover, the decreased room-temperature *ε*_r_ also explains the delayed polarization saturation behavior.


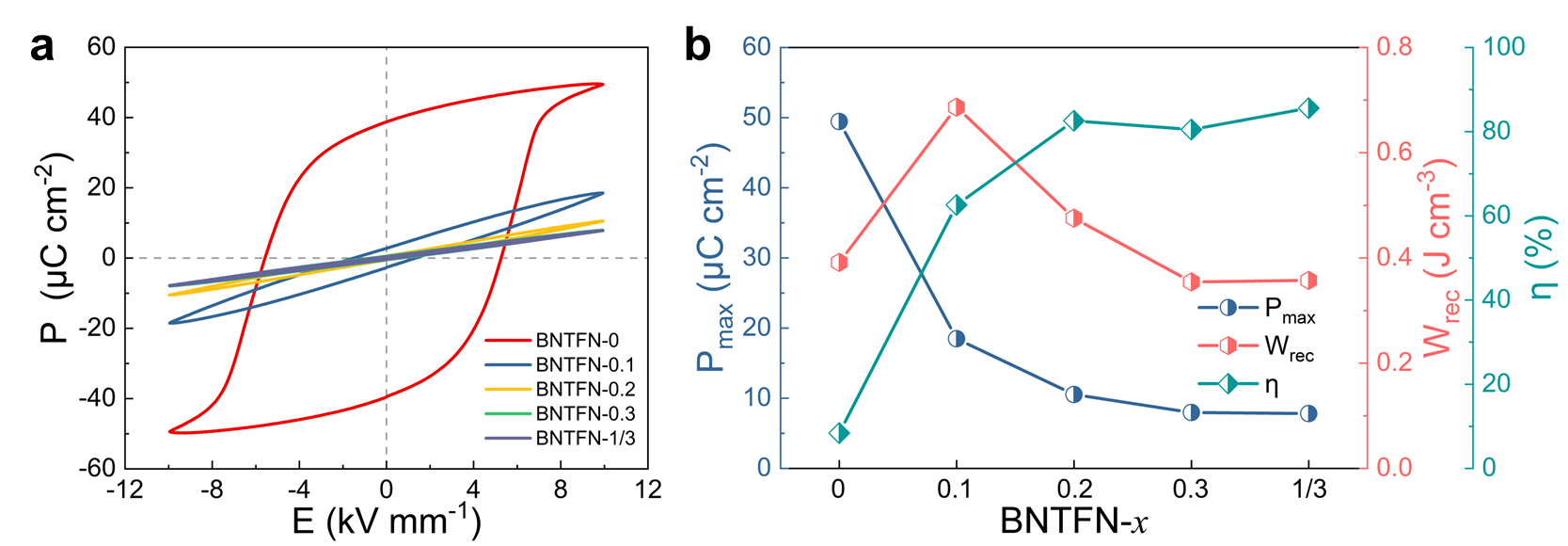


**Fig. S3** **a** The bipolar *P*-*E* loops of BNTFN-*x* (*x*=0, 0.1, 0.2, 0.3, 1/3) ceramics under 10 kV mm^-1^. **b** *P*_max_, *W*_rec_ and *η* under 10 kV mm^-1^ for BNTFN-*x* (*x*=0, 0.1, 0.2, 0.3, 1/3) ceramics

With the introduction of Fe^3+^ and Nb^5+^, the ferroelectric hysteresis loops gradually become slimer, presenting that the introduction of Fe^3+^ and Nb^5+^ can effectively break the long-range ferroelectric order and decrease energy loss. Furthermore, *P*_max_ under 10 kV mm^-1^ presents a similar downward trend to room-temperature *ε*_r_, which is one of the important contributions of polarization. Notably, the ultralow *P*_max_ and *W*_rec_ in BNTFN-*x* (*x* =0.1, 0.2, 0.3, 1/3) ceramics are mainly caused by the insufficient external electric fields, which can be largely improved by high enough electric fields.


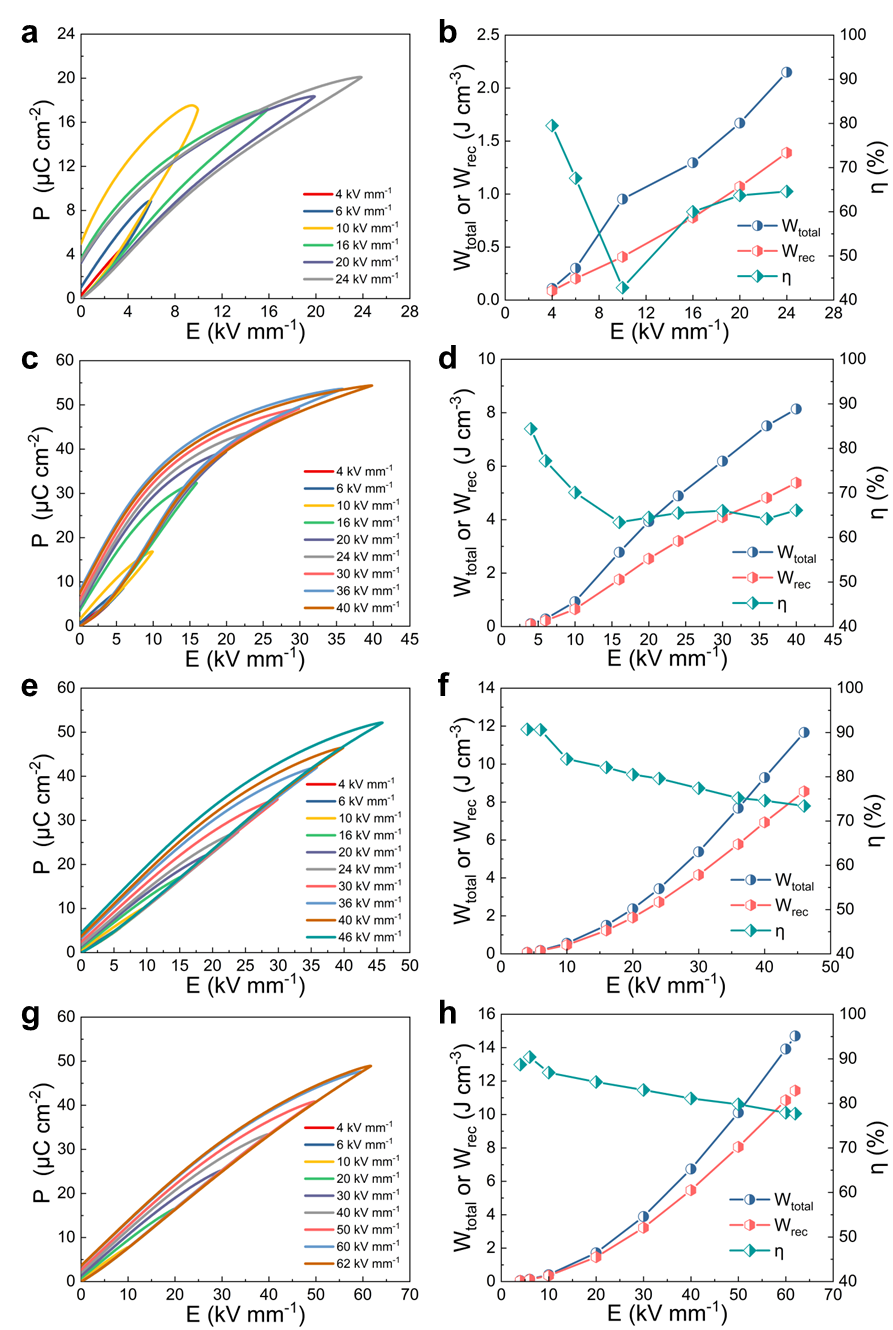


**Fig. S4** The *P*-*E* loops, and *W*_total_, *W*_rec_ and *η* from low electric field to *E*_b_ for **a-b** BNTFN-0 ceramics, **c-d** BNTFN-0.1 ceramics, **e-f** BNTFN-0.2 ceramics, and **g-h** BNTFN-0.3 ceramics


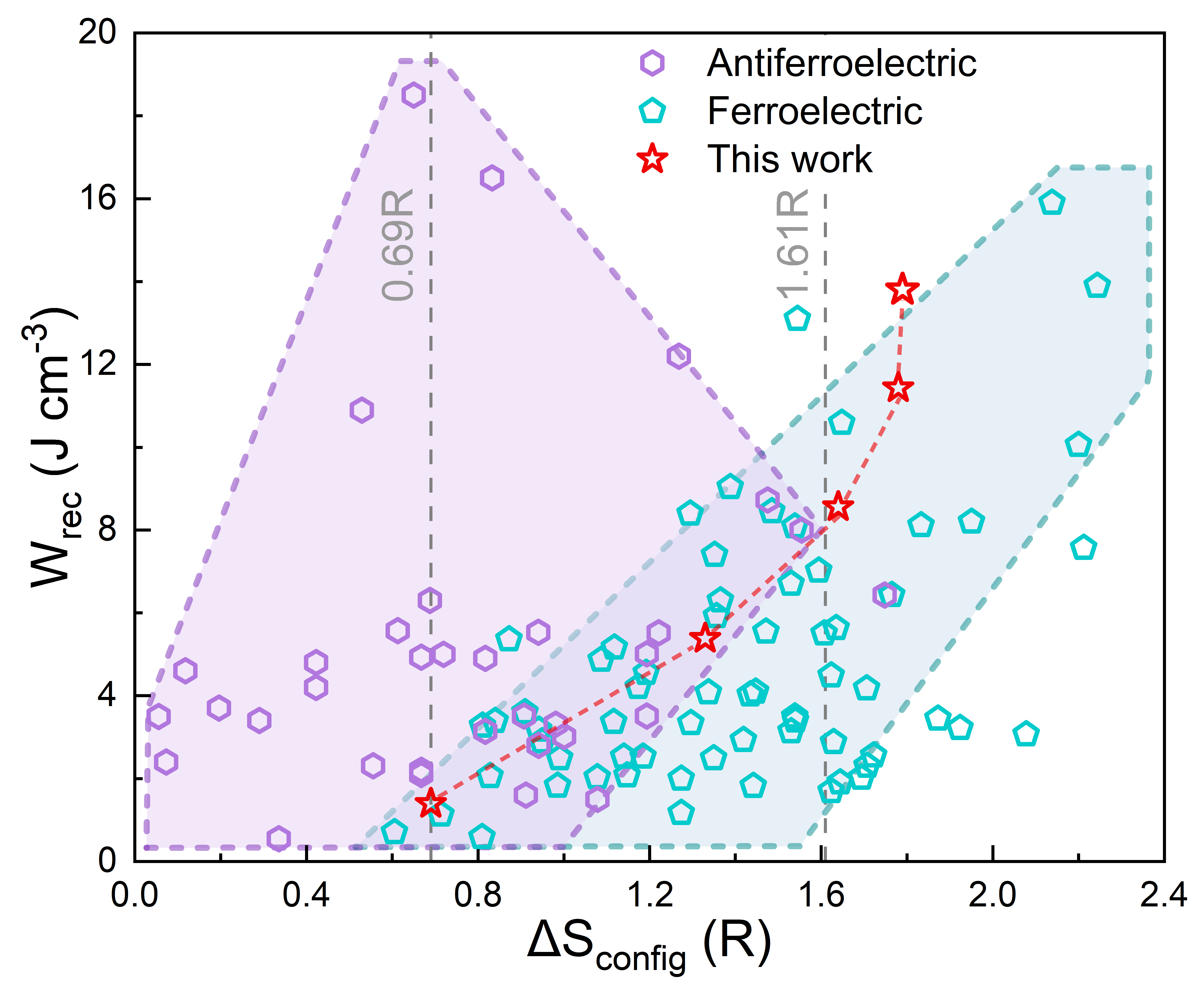


**Fig. S5** A comparison of *W*_rec_ and Δ*S*_config_ between the studied samples in this work and other reported lead-free relaxor ferroelectric and antiferroelectric ceramics


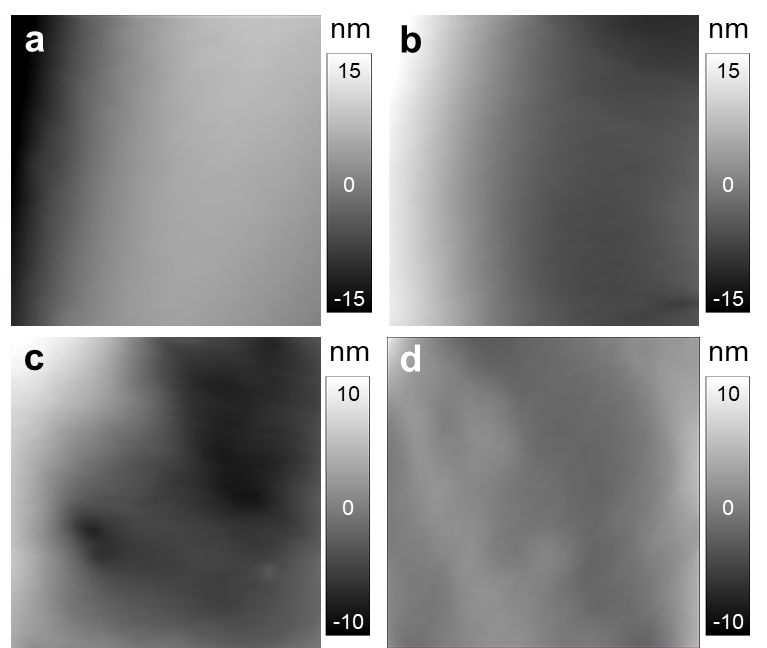


**Fig. S6** Out-of-plane PFM morphologies within one grain for **a** BNTFN-0, **b** BNTFN-0.1, **c** BNTFN-0.2, and **d** BNTFN-1/3 ceramics


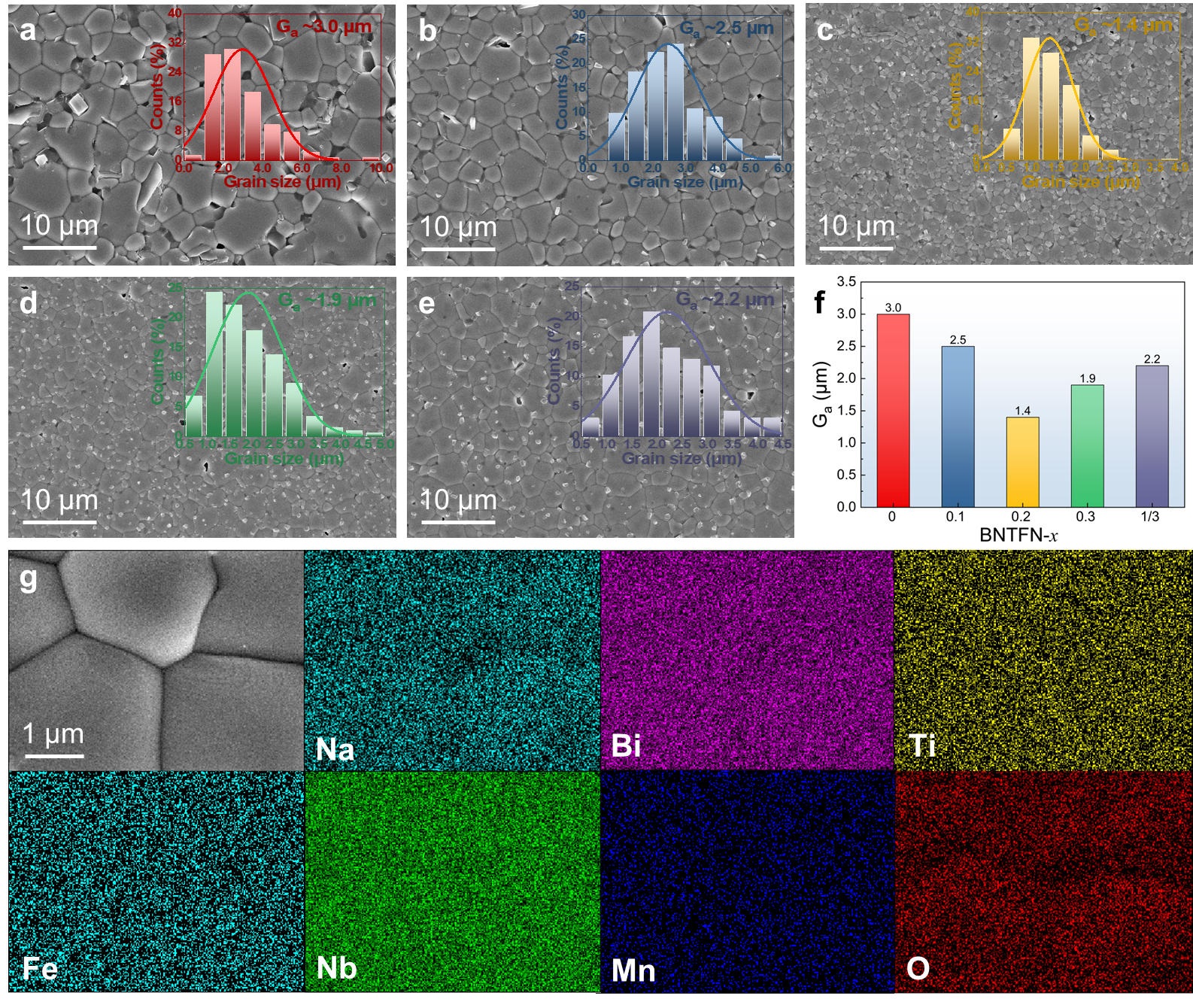


**Fig. S7** Thermal etched SEM morphology and grain size distribution for **a** BNTFN-0, **b** BNTFN-0.1, **c** BNTFN-0.2, **d** BNTFN-0.3, and **e** BNTFN-1/3 ceramics. **f** *G*_a_ for BNTFN-*x* (*x*=0, 0.1, 0.2, 0.3, 1/3) ceramics. **g** Element distribution maps of BNTFN-1/3 ceramic

The element distribution maps of BNTFN-1/3 ceramic are performed in adjacent grains with triangular grain boundaries. Not only a densely contact grain boundary structure but also a uniform element distribution between the grains and grain boundaries can be clearly observed in the studied sample, proving a good sample quality again.


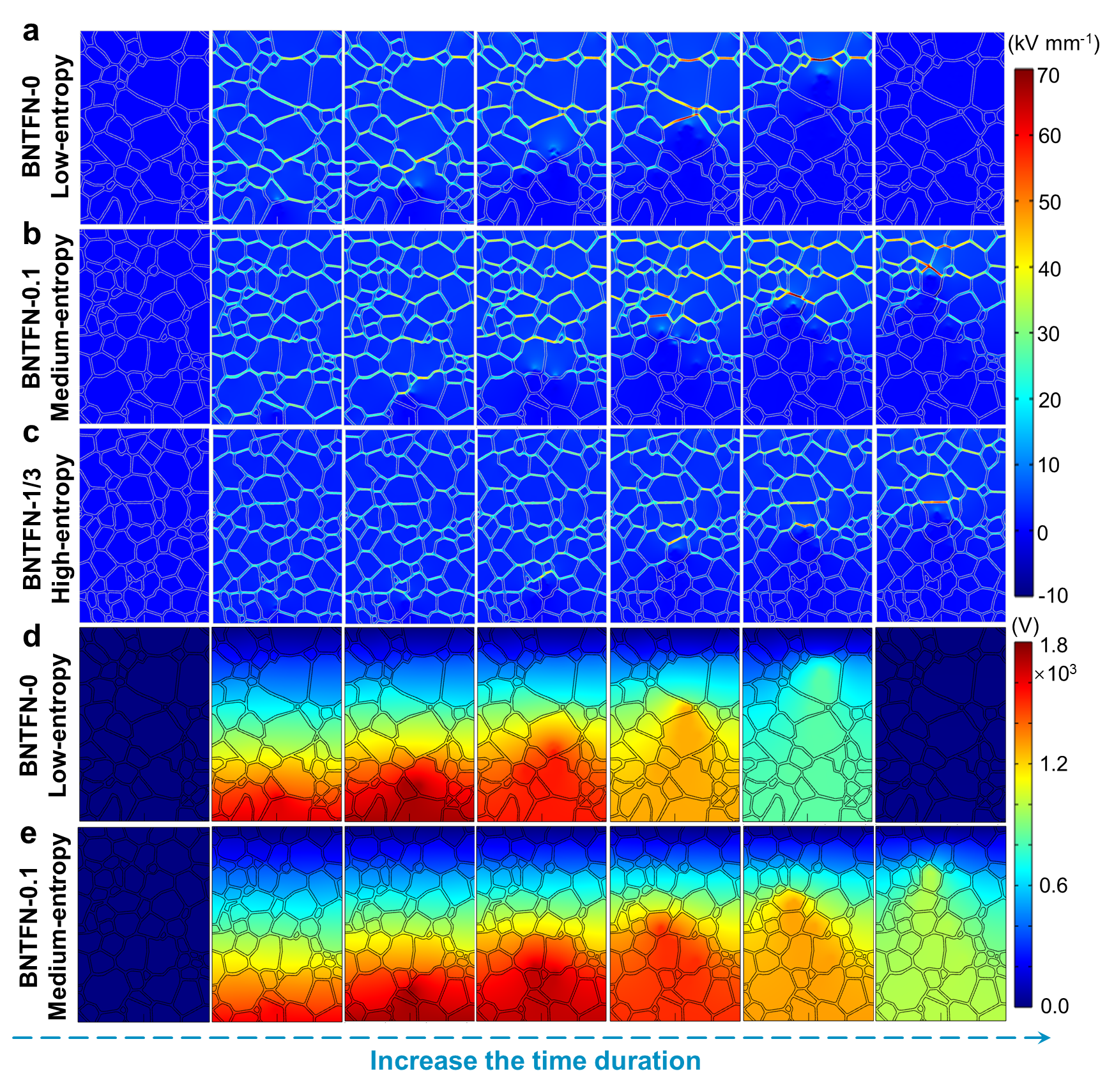


**Fig. S8** Electric field distribution for **a** BNTFN-0, **b** BNTFN-0.1, and **c** BNTFN-1/3 ceramics from low-entropy to high-entropy. Electric potential distribution for **d** BNTFN-0 and **e** BNTFN-0.1 ceramic


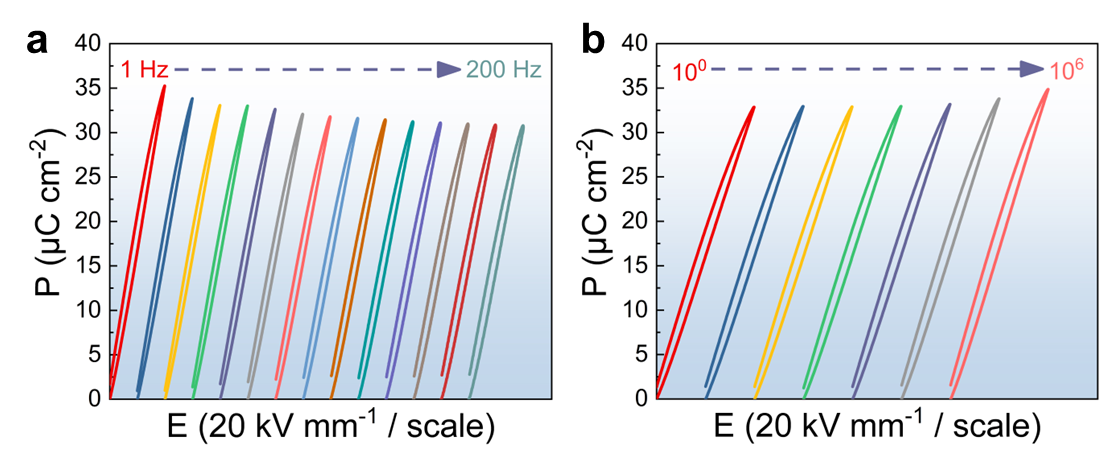


**Fig. S9** **a** Frequency-dependent *P*-*E* loops under 40 kV mm^-1^ for BNTFN-1/3 ceramic. **b** *P*-*E* loops as a function of the cycle number under 40 kV mm^-1^ for BNTFN-1/3 ceramic


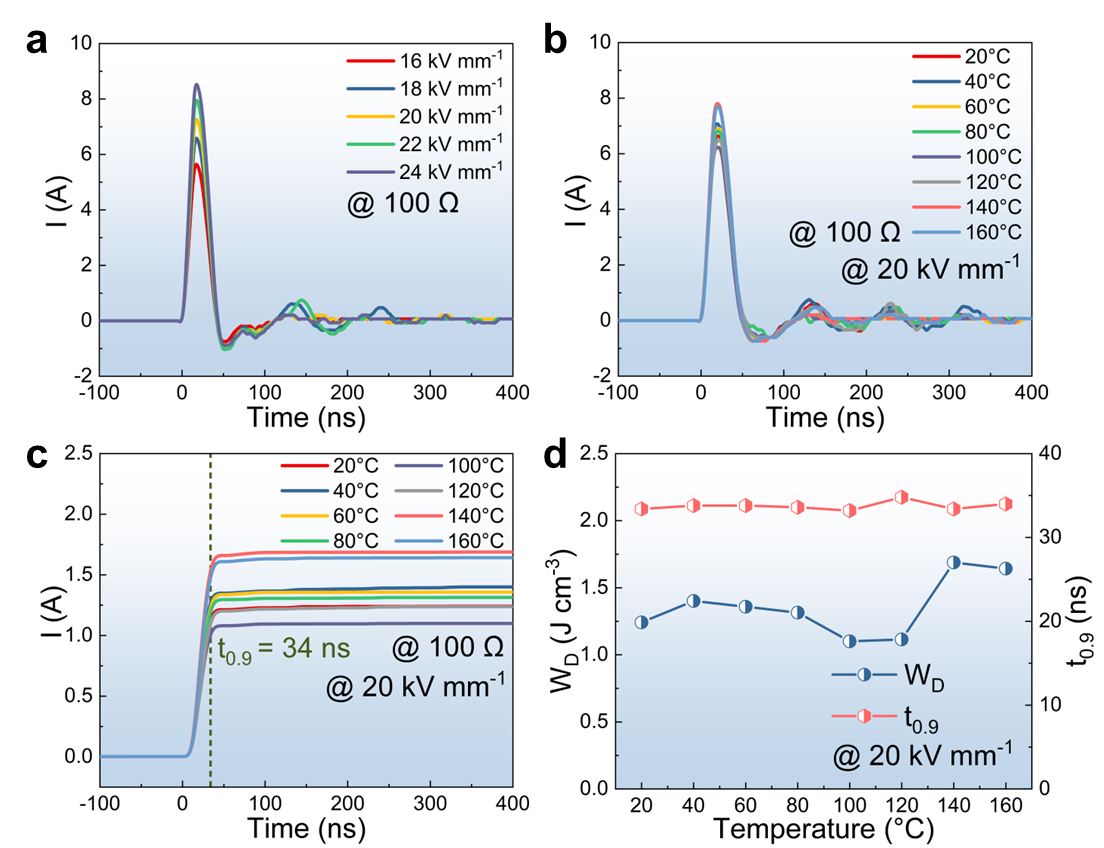


**Fig. S10** **a** Overdamped discharge waveforms for BNTFN-1/3 ceramic. **b** Overdamped discharge waveforms, **c** Calculated overdamped discharge density *W*_D_, and **d** *W*_D_ and *t*_0.9_ values at 20 kV mm^-1^ under different temperature for the BNTFN-1/3 ceramic (*R*_o_ =100 Ω)

The discharge energy density *W*_D_ can be calculated using the following formulas:

$W_{D}=\frac{R_{o}\int I_{(t)}^{2}dt}{V}$ (6)

where *V* is the sample volume.


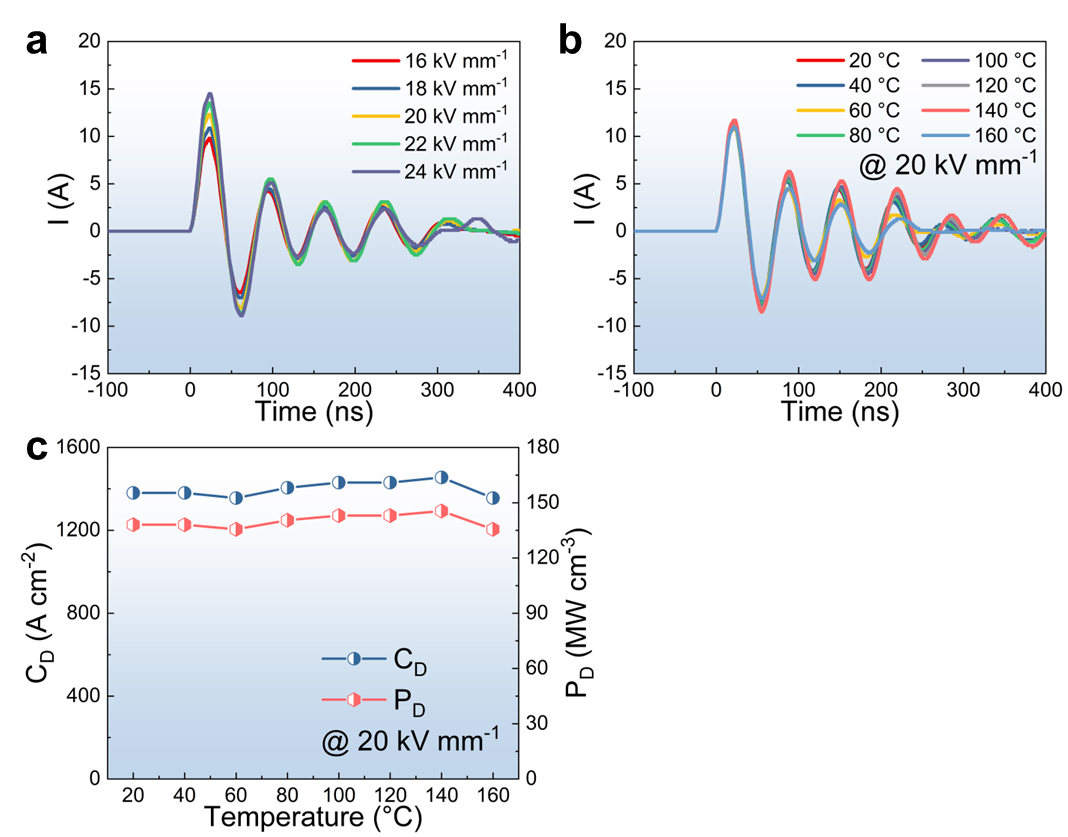


**Fig. S11 a** Underdamped discharge waveforms for BNTFN-1/3 ceramic. **b** Underdamped discharge waveforms, and **c** *C*_D_ and *P*_D_ values at 20 kV mm^-1^ under different temperatures

The current density *C*_D_ and power density *P*_D_ can be calculated using the following formulas:

$C_{D}=\frac{I_{max}}{S}$ (7)

$P_{D}=\frac{E\times I_{max}}{2S}$ (8)

where *S* is the electrode area.

**Table S1** Relevant references for the Fig. 2e,f.

| **Figure** | ***Ref.*** |
| --- | --- |
| Fig. 2e,f | [6-195] |

**References**

1. Z. Cai, X. Wang, W. Hong, B. Luo, Q. Zhao et al., Grain‐size–dependent dielectric properties in nanograin ferroelectrics. J. Am. Ceram. Soc. **101**(12), 5487-5496 (2018).

2. Z. Cai, X. Wang, B. Luo, W. Hong, L. Wu et al., Dielectric response and breakdown behavior of polymer-ceramic nanocomposites: The effect of nanoparticle distribution. Compos. Sci. Technol. **145**, 105-113 (2017).

3. K.M. Johnson, Variation of dielectric constant with voltage in ferroelectrics and its application to parametric devices. J. Appl. Phys. **33**(9), 2826-2831 (1962).

4. X. Dong, X. Li, X. Chen, Z. Tan, J. Wu et al., (1-*x*)[0.90NN-0.10Bi(Mg_2/3_Nb_1/3_)O_3_]-*x*(Bi_0.5_Na_0.5_)_0.7_Sr_0.3_TiO_3_ ceramics with core–shell structures: A pathway for simultaneously achieving high polarization and breakdown strength. Nano Energy **101**, 107577 (2022).

5. L. Padurariu, L. Curecheriu, V. Buscaglia, L. Mitoseriu, Field-dependent permittivity in nanostructured BaTiO_3_ ceramics: Modeling and experimental verification. Phys. Rev. B **85**(22), 224111 (2012).

6. W. Liu, J. Gao, Y. Zhao, S. Li, Significant enhancement of energy storage properties of BaTiO_3_-based ceramics by hybrid-doping. J. Alloys Compd. **843**, 155938 (2020).

7. D. Zhan, Q. Xu, D.-P. Huang, H.-X. Liu, W. Chen et al., Dielectric nonlinearity and electric breakdown behaviors of Ba_0.95_Ca_0.05_Zr_0.3_Ti_0.7_O_3_ ceramics for energy storage utilizations. J. Alloys Compd. **682**, 594-600 (2016).

8. Z. Shen, X. Wang, B. Luo, L. Li, BaTiO_3_–BiYbO_3_ perovskite materials for energy storage applications. J. Mater. Chem. A **3**(35), 18146-18153 (2015).

9. X. Yi, C. Ji, G. Chen, H. Yang, H. Yong et al., Wang, Effects of Sintering Method and BiAlO_3_ Dopant on Dielectric Relaxation and Energy Storage Properties of BaTiO_3_–BiYbO_3_ Ceramics. physica status solidi (a) **217**(2), 1900721 (2020).

10. T. Wang, L. Jin, C. Li, Q. Hu, X. Wei, Relaxor ferroelectric BaTiO_3_–Bi(Mg_2/3_Nb_1/3_)O_3_ ceramics for energy storage application. J. Am. Ceram. Soc. **98**(2), 559-566 (2015).

11. Z. Sun, L. Li, S. Yu, X. Kang, S. Chen, Energy storage properties and relaxor behavior of lead-free Ba_1−_*_x_*Sm_2_*_x_*_/3_Zr_0.15_Ti_0.85_O_3_ ceramics. Dalt. Trans. **46**(41), 14341-14347 (2017).

12. X. Jiang, H. Hao, S. Zhang, J. Lv, M. Cao et al., Enhanced energy storage and fast discharge properties of BaTiO_3_ based ceramics modified by Bi(Mg_1/2_Zr_1/2_)O_3_. J. Eur. Ceram. Soc. **39**(4), 1103-1109 (2019).

13. X. Liu, H. Yang, F. Yan, Y. Qin, Y. Lin et al., Enhanced energy storage properties of BaTiO_3_-Bi_0.5_Na_0.5_TiO_3_ lead-free ceramics modified by SrY_0.5_Nb_0.5_O_3_. J. Alloys Compd. **778**, 97-104 (2019).

14. C. Zhu, Z. Cai, L. Li, X. Wang, High energy density, high efficiency and excellent temperature stability of lead free Mn–doped BaTiO_3_–Bi(Mg_1/2_Zr_1/2_)O_3_ ceramics sintered in a reducing atmosphere. J. Alloys Compd. **816**, 152498 (2020).

15. L. Zhang, L.-X. Pang, W.-B. Li, D. Zhou, Extreme high energy storage efficiency in perovskite structured (1-*x*)(Ba_0.8_Sr_0.2_)TiO_3_-xBi(Zn_2/3_Nb_1/3_)O_3_ (0.04≤*x*≤0.16) ceramics. J. Eur. Ceram. Soc. **40**(8), 3343-3347 (2020).

16. W.-B. Li, D. Zhou, L.-X. Pang, Enhanced energy storage density by inducing defect dipoles in lead free relaxor ferroelectric BaTiO_3_-based ceramics. Appl. Phys. Lett. **110**(13), 132902 (2017).

17. Y. Li, J. Bian, Effects of reoxidation on the dielectric and energy storage properties of Ce-doped (Ba,Sr)TiO_3_ ceramics prepared by hot-pressed sintering. J. Eur. Ceram. Soc. **40**(15), 5441-5449 (2020).

18. Q. Hu, L. Jin, T. Wang, C. Li, Z. Xing et al., Dielectric and temperature stable energy storage properties of 0.88BaTiO_3_–0.12Bi (Mg_1/2_Ti_1/2_)O_3_ bulk ceramics. J. Alloys Compd. **640**, 416-420 (2015).

19. A. Jain, Y. Wang, N. Wang, F. Wang, Critical role of CuO doping on energy storage performance and electromechanical properties of Ba_0.8_Sr_0.1_Ca_0.1_Ti_0.9_Zr_0.1_O_3_ ceramics. Ceram. Int. **46**(11), 18800-18812 (2020).

20. G. Liu, Y. Li, M. Shi, L. Yu, P. Chen et al., An investigation of the dielectric energy storage performance of Bi(Mg_2/3_Nb_1/3_)O_3_-modifed BaTiO_3_ Pb-free bulk ceramics with improved temperature/frequency stability. Ceram. Int. **45**(15), 19189-19196 (2019).

21. H. Yang, F. Yan, Y. Lin, T. Wang, Enhanced energy storage properties of Ba_0.4_Sr_0.6_TiO_3_ lead-free ceramics with Bi_2_O_3_-B_2_O_3_-SiO_2_ glass addition. J. Eur. Ceram. Soc. **38**(4), 1367-1373 (2018).

22. W.-B. Li, D. Zhou, L.-X. Pang, Structure and energy storage properties of Mn-doped (Ba,Sr)TiO_3_–MgO composite ceramics. J. Mater. Sci.: Mater. Electron. **28**(12), 8749-8754 (2017).

23. Z. Dai, J. Xie, X. Fan, X. Ding, W. Liu et al., Enhanced energy storage properties and stability of Sr(Sc_0.5_Nb_0.5_)O_3_ modified 0.65BaTiO_3_-0.35Bi_0.5_Na_0.5_TiO_3_ ceramics. Chem. Eng. J. **397**, 125520 (2020).

24. Y. Huang, C. Zhao, B. Wu, J. Wu, Multifunctional BaTiO_3_-based relaxor ferroelectrics toward excellent energy storage performance and electrostrictive strain benefiting from crossover region. ACS Appl. Mater. Interfaces **12**(21), 23885-23895 (2020).

25. M. Zhou, R. Liang, Z. Zhou, X. Dong, Combining high energy efficiency and fast charge-discharge capability in novel BaTiO_3_-based relaxor ferroelectric ceramic for energy-storage. Ceram. Int. **45**(3), 3582-3590 (2019).

26. M. Zhou, R. Liang, Z. Zhou, X. Dong, Novel BaTiO_3_-based lead-free ceramic capacitors featuring high energy storage density, high power density, and excellent stability. J. Mater. Chem. C **6**(31), 8528-8537 (2018).

27. F. Si, B. Tang, Z. Fang, H. Li, S. Zhang, A new type of BaTiO_3_-based ceramics with Bi(Mg_1/2_Sn_1/2_)O_3_ modification showing improved energy storage properties and pulsed discharging performances. J. Alloys Compd. **819**, 153004 (2020).

28. Q. Wang, P.-M. Gong, C.-M. Wang, High recoverable energy storage density and large energy efficiency simultaneously achieved in BaTiO_3_–Bi(Zn_1/2_Zr_1/2_)O_3_ relaxor ferroelectrics. Ceram. Int. **46**(14), 22452-22459 (2020).

29. X. Chen, X. Li, J. Sun, C. Sun, J. Shi et al., Achieving ultrahigh energy storage density and energy efficiency simultaneously in barium titanate based ceramics. Appl. Phys. A **126**(2), 1-8 (2020).

30. A. Jain, Y. Wang, H. Guo, Microstructural properties and ultrahigh energy storage density in Ba_0.9_Ca_0.1_TiO_3_–NaNb_0.85_Ta_0.15_O_3_ relaxor ceramics. Ceram. Int. **46**(15), 24333-24346 (2020).

31. F. Si, B. Tang, Z. Fang, H. Li, S. Zhang, Enhanced energy storage and fast charge-discharge properties of (1-*x*)BaTiO_3_-*x*Bi(Ni_1/2_Sn_1/2_)O_3_ relaxor ferroelectric ceramics. Ceram. Int. **45**(14), 17580-17590 (2019).

32. Q. Yuan, G. Li, F.-Z. Yao, S.-D. Cheng, Y. Wang et al., Simultaneously achieved temperature-insensitive high energy density and efficiency in domain engineered BaTiO_3_-Bi (Mg_0.5_Zr_0.5_)O_3_ lead-free relaxor ferroelectrics. Nano Energy **52**, 203-210 (2018).

33. X. Li, X. Chen, J. Sun, M. Zhou, H. Zhou, Novel lead-free ceramic capacitors with high energy density and fast discharge performance. Ceram. Int. **46**(3), 3426-3432 (2020).

34. Y. Wang, S. Gao, T. Wang, J. Liu, D. Li et al., Structure, dielectric properties of novel Ba(Zr,Ti)O_3_ based ceramics for energy storage application. Ceram. Int. **46**(8), 12080-12087 (2020).

35. X. Chen, X. Li, J. Sun, C. Sun, J. Shi et al., Simultaneously achieving ultrahigh energy storage density and energy efficiency in barium titanate based ceramics. Ceram. Int. **46**(3), 2764-2771 (2020).

36. Z. Dai, J. Xie, W. Liu, X. Wang, L. Zhang et al., Effective strategy to achieve excellent energy storage properties in lead-free BaTiO_3_-based bulk ceramics. ACS Appl. Mater. Interfaces **12**(27), 30289-30296 (2020).

37. Z.-G. Liu, M.-D. Li, Z.-H. Tang, X.-G. Tang, Enhanced energy storage density and efficiency in lead-free Bi(Mg_1/2_Hf_1/2_)O_3_-modified BaTiO_3_ ceramics. Chem. Eng. J. **418**, 129379 (2021).

38. G. Liu, Y. Li, B. Guo, M. Tang, Q. Li et al., Ultrahigh dielectric breakdown strength and excellent energy storage performance in lead-free barium titanate-based relaxor ferroelectric ceramics via a combined strategy of composition modification, viscous polymer processing, and liquid-phase sintering. Chem. Eng. J. **398**, 125625 (2020).

39. Z.-G. Liu, Z.-H. Tang, S.-C. Hu, D.-J. Yao, F. Sun et al., Excellent energy storage density and efficiency in lead-free Sm-doped BaTiO_3_–Bi(Mg_0.5_Ti_0.5_)O_3_ ceramics. J. Mater. Chem. C **8**(38), 13405-13414 (2020).

40. D. Hu, Z. Pan, X. Tan, F. Yang, J. Ding et al., Optimization the energy density and efficiency of BaTiO_3_-based ceramics for capacitor applications. Chem. Eng. J. **409**, 127375 (2021).

41. Y. Li, Y. Liu, M. Tang, J. Lv, F. Chen et al., Energy storage performance of BaTiO_3_-based relaxor ferroelectric ceramics prepared through a two-step process. Chem. Eng. J. **419**, 129673 (2021).

42. X. Dong, X. Li, X. Chen, J. Wu, H. Zhou, Simultaneous enhancement of polarization and breakdown strength in lead-free BaTiO_3_-based ceramics. Chem. Eng. J. **409**, 128231 (2021).

43. Q. Hu, Y. Tian, Q. Zhu, J. Bian, L. Jin et al., Achieve ultrahigh energy storage performance in BaTiO_3_–Bi(Mg_1/2_Ti_1/2_)O_3_ relaxor ferroelectric ceramics via nano-scale polarization mismatch and reconstruction. Nano Energy **67**, 104264 (2020).

44. W. Huang, Y. Chen, X. Li, G. Wang, N. Liu et al., Ultrahigh recoverable energy storage density and efficiency in barium strontium titanate-based lead-free relaxor ferroelectric ceramics. Appl. Phys. Lett. **113**(20), 203902 (2018).

45. H. Yang, Z. Lu, L. Li, W. Bao, H. Ji et al., Novel BaTiO_3_-based, Ag/Pd-compatible lead-free relaxors with superior energy storage performance. ACS Appl. Mater. Interfaces **12**(39), 43942-43949 (2020).

46. W. Wang, L. Zhang, R. Jing, Q. Hu, D. Alikin et al., Enhancement of energy storage performance in lead-free barium titanate-based relaxor ferroelectrics through a synergistic two-step strategy design. Chem. Eng. J. **434**, 134678 (2022).

47. W. Huang, Y. Chen, X. Li, G. Wang, J. Xia et al., Superior energy storage performances achieved in (Ba,Sr)TiO_3_-based bulk ceramics through composition design and Core-shell structure engineering. Chem. Eng. J. **444**, 135523 (2022).

48. L. Li, X. Yu, H. Cai, Q. Liao, Y. Han et al., Preparation and dielectric properties of BaCu(B_2_O_5_)-doped SrTiO_3_-based ceramics for energy storage. Mater. Sci. Eng.: B **178**(20), 1509-1514 (2013).

49. H. Yang, F. Yan, Y. Lin, T. Wang, L. He et al., A lead free relaxation and high energy storage efficiency ceramics for energy storage applications. J. Alloys Compd. **710**, 436-445 (2017).

50. J. Wang, H. Fan, B. Hu, H. Jiang, Enhanced energy-storage performance and temperature-stable dielectric properties of (1−*x*)(0.94Na_0.5_Bi_0.5_TiO_3_–0.06BaTiO_3_)–*x*Na_0.73_Bi_0.09_NbO_3_ ceramics. J. Mater. Sci.: Mater. Electron. **30**(3), 2479-2488 (2019).

51. H. Wang, H. Yuan, X. Li, F. Zeng, K. Wu et al., Enhanced energy density and discharged efficiency of lead-free relaxor (1-*x*)[(Bi_0.5_Na_0.5_)_0.94_Ba_0.06_]_0.98_La_0.02_TiO_3_-*x*KNb_0.6_Ta_0.4_O_3_ ceramic capacitors. Chem. Eng. J. **394**, 124879 (2020).

52. P. Zhao, B. Tang, F. Si, C. Yang, H. Li et al., Novel Ca doped Sr_0.7_Bi_0.2_TiO_3_ lead-free relaxor ferroelectrics with high energy density and efficiency. J. Eur. Ceram. Soc. **40**(5), 1938-1946 (2020).

53. Z. Chen, X. Bu, B. Ruan, J. Du, P. Zheng et al., Simultaneously achieving high energy storage density and efficiency under low electric field in BiFeO_3_-based lead-free relaxor ferroelectric ceramics. J. Eur. Ceram. Soc. **40**(15), 5450-5457 (2020).

54. R. Kang, Z. Wang, W. Liu, L. He, X. Zhu et al., Domain engineered lead-free ceramics with large energy storage density and ultra-high efficiency under low electric fields. ACS Appl. Mater. Interfaces **13**(21), 25143-25152 (2021).

55. X. Kong, L. Yang, Z. Cheng, S. Zhang, Bi‐modified SrTiO_3_‐based ceramics for high‐temperature energy storage applications. J. Am. Ceram. Soc. **103**(3), 1722-1731 (2020).

56. Y. Huang, Q. Guo, H. Hao, H. Liu, S. Zhang, Tailoring properties of (Bi_0.51_Na_0.47_)TiO_3_ based dielectrics for energy storage applications. J. Eur. Ceram. Soc. **39**(15), 4752-4760 (2019).

57. Y. Pu, W. Wang, X. Guo, R. Shi, M. Yang et al., Enhancing the energy storage properties of Ca_0.5_Sr_0.5_TiO_3_-based lead-free linear dielectric ceramics with excellent stability through regulating grain boundary defects. J. Mater. Chem. C **7**(45), 14384-14393 (2019).

58. L. Yang, X. Kong, Z. Cheng, S. Zhang, Ultra-high energy storage performance with mitigated polarization saturation in lead-free relaxors. J. Mater. Chem. A **7**(14), 8573-8580 (2019).

59. M. Zhou, R. Liang, Z. Zhou, X. Dong, Achieving ultrahigh energy storage density and energy efficiency simultaneously in sodium niobate-based lead-free dielectric capacitors via microstructure modulation. Inorg. Chem. Fron. **6**(8), 2148-2157 (2019).

60. X. Zhang, D. Hu, Z. Pan, X. Lv, Z. He et al., Enhancement of recoverable energy density and efficiency of lead-free relaxor-ferroelectric BNT-based ceramics. Chem. Eng. J. **406**, 126818 (2021).

61. X. Qiao, F. Zhang, D. Wu, B. Chen, X. Zhao et al., Superior comprehensive energy storage properties in Bi_0.5_Na_0.5_TiO_3_-based relaxor ferroelectric ceramics. Chem. Eng. J. **388**, 124158 (2020).

62. T. Wei, K. Liu, P. Fan, D. Lu, B. Ye et al., Novel NaNbO_3_–Sr_0.7_Bi_0.2_TiO_3_ lead-free dielectric ceramics with excellent energy storage properties. Ceram. Int. **47**(3), 3713-3719 (2021).

63. F. Yan, K. Huang, T. Jiang, X. Zhou, Y. Shi et al., Significantly enhanced energy storage density and efficiency of BNT-based perovskite ceramics via A-site defect engineering. Energy Storage Mater. **30**, 392-400 (2020).

64. H. Ji, D. Wang, W. Bao, Z. Lu, G. Wang et al., Ultrahigh energy density in short-range tilted NBT-based lead-free multilayer ceramic capacitors by nanodomain percolation. Energy Storage Mater. **38**, 113-120 (2021).

65. H. Chen, J. Shi, X. Chen, C. Sun, F. Pang et al., Excellent energy storage properties and stability of NaNbO_3_–Bi(Mg_0.5_Ta_0.5_)O_3_ ceramics by introducing (Bi_0.5_Na_0.5_)_0.7_Sr_0.3_TiO_3_. J. Mater. Chem. A **9**(8), 4789-4799 (2021).

66. H. Qi, A. Xie, A. Tian, R. Zuo, Superior energy‐storage capacitors with simultaneously giant energy density and efficiency using nanodomain engineered BiFeO_3_‐BaTiO_3_‐NaNbO_3_ lead‐free bulk ferroelectrics. Adv. Energy Mater. **10**(6), 1903338 (2020).

67. N. Luo, K. Han, F. Zhuo, C. Xu, G. Zhang et al., Aliovalent A-site engineered AgNbO_3_ lead-free antiferroelectric ceramics toward superior energy storage density. J. Mater. Chem. A **7**(23), 14118-14128 (2019).

68. J. Gao, L. Zhao, Q. Liu, X. Wang, S. Zhang et al., Antiferroelectric‐ferroelectric phase transition in lead‐free AgNbO_3_ ceramics for energy storage applications. J. Am. Ceram. Soc. **101**(12), 5443-5450 (2018).

69. N. Luo, K. Han, M.J. Cabral, X. Liao, S. Zhang et al., Constructing phase boundary in AgNbO_3_ antiferroelectrics: pathway simultaneously achieving high energy density and efficiency. Nature Commun. **11**, 4824 (2020).

70. S. Mao, N. Luo, K. Han, Q. Feng, X. Chen et al., Effect of Lu doping on the structure, electrical properties and energy storage performance of AgNbO_3_ antiferroelectric ceramics. J. Mater. Sci.: Mater. Electron. **31**(10), 7731-7741 (2020).

71. A. Song, J. Song, Y. Lv, L. Liang, J. Wang et al., Energy storage performance in BiMnO_3_-modified AgNbO_3_ anti-ferroelectric ceramics. Mater. Lett. **237**, 278-281 (2019).

72. J. Gao, Y. Zhang, L. Zhao, K.-Y. Lee, Q. Liu et al., Enhanced antiferroelectric phase stability in La-doped AgNbO_3_: perspectives from the microstructure to energy storage properties. J. Mater. Chem. A **7**(5), 2225-2232 (2019).

73. P. Ren, D. Ren, L. Sun, F. Yan, S. Yang et al., Grain size tailoring and enhanced energy storage properties of two-step sintered Nd^3+^-doped AgNbO_3_. J. Eur. Ceram. Soc. **40**(13), 4495-4502 (2020).

74. Y. Tian, L. Jin, H. Zhang, Z. Xu, X. Wei et al., High energy density in silver niobate ceramics. J. Mater. Chem. A **4**(44), 17279-17287 (2016).

75. Y. Xu, Y. Guo, Q. Liu, G. Wang, J. Bai et al., High energy storage properties of lead-free Mn-doped (1-*x*)AgNbO_3_-*x*Bi_0.5_Na_0.5_TiO_3_ antiferroelectric ceramics. J. Eur. Ceram. Soc. **40**(1), 56-62 (2020).

76. C. Xu, Z. Fu, Z. Liu, L. Wang, S. Yan et al., La/Mn codoped AgNbO_3_ lead-free antiferroelectric ceramics with large energy density and power density. ACS Sustainable Chem. Eng. **6**(12), 16151-16159 (2018).

77. N. Luo, K. Han, L. Liu, B. Peng, X. Wang et al., Lead‐free Ag_1−3_*_x_*La*_x_*NbO_3_ antiferroelectric ceramics with high‐energy storage density and efficiency. J. Am. Ceram. Soc. **102**(8), 4640-4647 (2019).

78. L. Zhao, Q. Liu, S. Zhang, J.-F. Li, Lead-free AgNbO_3_ anti-ferroelectric ceramics with an enhanced energy storage performance using MnO_2_ modification. J. Mater. Chem. C **4**(36), 8380-8384 (2016).

79. L. Zhao, Q. Liu, J. Gao, S. Zhang, J.F. Li, Lead‐free antiferroelectric silver niobate tantalate with high energy storage performance. Adv. Mater. **29**(31), 1701824 (2017).

80. J. Gao, Q. Liu, J. Dong, X. Wang, S. Zhang et al., Local structure heterogeneity in Sm-doped AgNbO_3_ for improved energy-storage performance. ACS Appl. Mater. Interfaces **12**(5), 6097-6104 (2020).

81. Z. Lu, W. Bao, G. Wang, S.-K. Sun, L. Li et al., Mechanism of enhanced energy storage density in AgNbO_3_-based lead-free antiferroelectrics. Nano Energy **79**, 105423 (2021).

82. Y. Tian, L. Jin, H. Zhang, Z. Xu, X. Wei et al., Phase transitions in bismuth-modified silver niobate ceramics for high power energy storage. J. Mater. Chem. A **5**(33), 17525-17531 (2017).

83. K. Han, N. Luo, S. Mao, F. Zhuo, X. Chen et al., Realizing high low-electric-field energy storage performance in AgNbO_3_ ceramics by introducing relaxor behaviour. J. Materiomics **5**(4), 597-605 (2019).

84. S. Li, H. Nie, G. Wang, C. Xu, N. Liu et al., Significantly enhanced energy storage performance of rare-earth-modified silver niobate lead-free antiferroelectric ceramics via local chemical pressure tailoring. J. Mater. Chem. C **7**(6), 1551-1560 (2019).

85. Z. Yan, D. Zhang, X. Zhou, H. Qi, H. Luo et al., Silver niobate based lead-free ceramics with high energy storage density. J. Mater. Chem. A **7**(17), 10702-10711 (2019).

86. L. Zhao, J. Gao, Q. Liu, S. Zhang, J.-F. Li, Silver niobate lead-free antiferroelectric ceramics: enhancing energy storage density by B-site doping. ACS Appl. Mater. Interfaces **10**(1), 819-826 (2018).

87. K. Han, N. Luo, S. Mao, F. Zhuo, L. Liu et al., Ultrahigh energy-storage density in A-/B-site co-doped AgNbO_3_ lead-free antiferroelectric ceramics: insight into the origin of antiferroelectricity, J. Mater. Chem. A **7**(46), 26293-26301 (2019).

88. S. Li, T. Hu, H. Nie, Z. Fu, C. Xu et al., Giant energy density and high efficiency achieved in silver niobate-based lead-free antiferroelectric ceramic capacitors via domain engineering. Energy Storage Mater. **34**, 417-426 (2021).

89. W. Chao, T. Yang, Y. Li, Z. Liu, Enhanced energy storage density in Ca and Ta co‐doped AgNbO_3_ antiferroelectric ceramics. J. Am. Ceram. Soc. **103**(12), 7283-7290 (2020).

90. W. Chao, J. Gao, T. Yang, Y. Li, Excellent energy storage performance in La and Ta co-doped AgNbO_3_ antiferroelectric ceramics. J. Eur. Ceram. Soc. **41**(15), 7670-7677 (2021).

91. J. Li, Y. Tian, Y. Lan, L. Jin, C. Chen et al., Silver deficiency effect on dielectric properties and energy storage performance of AgNbO_3_ ceramics. Ceram. Int. **47**(18), 26178-26184 (2021).

92. P. Shi, X. Wang, X. Lou, C. Zhou, Q. Liu et al., Significantly enhanced energy storage properties of Nd^3+^ doped AgNbO_3_ lead-free antiferroelectric ceramics. J. Alloys Compd. **877**, 160162 (2021).

93. N. Luo, X. Tang, K. Han, L. Ma, Z. Chen et al., Silver stoichiometry engineering: an alternative way to improve energy storage density of AgNbO_3_-based antiferroelectric ceramics. J. Mater. Res. **36**(5), 1067-1075 (2021).

94. Y. Xu, Y. Guo, Q. Liu, Y. Yin, J. Bai et al., Enhanced energy density in Mn-doped (1-*x*)AgNbO_3_-*x*CaTiO_3_ lead-free antiferroelectric ceramics. J. Alloys Compd. **821**, 153260 (2020).

95. N. Liu, R. Liang, Z. Zhou, X. Dong, Designing lead-free bismuth ferrite-based ceramics learning from relaxor ferroelectric behavior for simultaneous high energy density and efficiency under low electric field. J. Mater. Chem. C **6**(38), 10211-10217 (2018).

96. D. Zheng, R. Zuo, Enhanced energy storage properties in La(Mg_1/2_Ti_1/2_)O_3_-modified BiFeO_3_-BaTiO_3_ lead-free relaxor ferroelectric ceramics within a wide temperature range. J. Eur. Ceram. Soc. **37**(1), 413-418 (2017).

97. S. Dabas, M. Kumar, P. Chaudhary, O. Thakur, Enhanced magneto-electric coupling and energy storage analysis in Mn-modified lead free BiFeO_3_-BaTiO_3_ solid solutions. J. Appl. Phys. **126**(13), 134102 (2019).

98. D. Wang, Z. Fan, W. Li, D. Zhou, A. Feteira et al., High energy storage density and large strain in Bi (Zn_2/3_Nb_1/3_)O_3_-doped BiFeO_3_–BaTiO_3_ ceramics. ACS Appl. Energy Mater. **1**(8), 4403-4412 (2018).

99. F. Akram, J. Kim, S.A. Khan, A. Zeb, H.G. Yeo et al., Less temperature-dependent high dielectric and energy-storage properties of eco-friendly BiFeO_3_–BaTiO_3_-based ceramics. J. Alloys Compd. **818**, 152878 (2020).

100. D. Zheng, R. Zuo, D. Zhang, Y. Li, Novel BiFeO_3_–BaTiO_3_–Ba(Mg_1/3_Nb_2/3_)O_3_ lead‐free relaxor ferroelectric ceramics for energy‐storage capacitors. J. Am. Ceram. Soc. **98**(9), 2692-2695 (2015).

101. N. Liu, R. Liang, X. Zhao, C. Xu, Z. Zhou et al., Novel bismuth ferrite‐based lead‐free ceramics with high energy and power density. J. Am. Ceram. Soc. **101**(8), 3259-3265 (2018).

102. Z. Lu, G. Wang, W. Bao, J. Li, L. Li et al., Superior energy density through tailored dopant strategies in multilayer ceramic capacitors. Energy Environ. Sci. **13**(9), 2938-2948 (2020).

103. Z. Chen, X. Bai, H. Wang, J. Du, W. Bai et al., Achieving high-energy storage performance in 0.67Bi_1-_*_x_*Sm*_x_*FeO_3_-0.33BaTiO_3_ lead-free relaxor ferroelectric ceramics. Ceram. Int. **46**(8), 11549-11555 (2020).

104. H. Yang, H. Qi, R. Zuo, Enhanced breakdown strength and energy storage density in a new BiFeO_3_-based ternary lead-free relaxor ferroelectric ceramic. J. Eur. Ceram. Soc. **39**(8), 2673-2679 (2019).

105. G. Wang, Z. Lu, H. Yang, H. Ji, A. Mostaed et al., Fatigue resistant lead-free multilayer ceramic capacitors with ultrahigh energy density. J. Mater. Chem. A **8**(22), 11414-11423 (2020).

106. H. Sun, X. Wang, Q. Sun, X. Zhang, Z. Ma et al., Large energy storage density in BiFeO_3_-BaTiO_3_-AgNbO_3_ lead-free relaxor ceramics. J. Eur. Ceram. Soc. **40**(8), 2929-2935 (2020).

107. X. Bai, Z. Chen, P. Zheng, W. Bai, J. Zhang et al., High recoverable energy storage density in nominal (0.67-*x*)BiFeO_3_-0.33BaTiO_3_-*x*BaBi_2_Nb_2_O_9_ lead-free composite ceramics. Ceram. Int. **47**(16), 23116-23123 (2021).

108. M. Shiga, M. Hagiwara, S. Fujihara, (Bi_1/2_K_1/2_)TiO_3_–SrTiO_3_ solid-solution ceramics for high-temperature capacitor applications. Ceram. Int. **46**(8), 10242-10249 (2020).

109. F. Li, T. Jiang, J. Zhai, B. Shen, H. Zeng, Exploring novel bismuth-based materials for energy storage applications. J. Mater. Chem. C **6**(30), 7976-7981 (2018).

110. F. Li, X. Hou, T. Li, R. Si, C. Wang et al., Fine-grain induced outstanding energy storage performance in novel Bi_0.5_K_0.5_TiO_3_–Ba(Mg_1/3_Nb_2/3_)O_3_ ceramics via a hot-pressing strategy. J. Mater. Chem. C **7**(39), 12127-12138 (2019).

111. F. Li, R. Si, T. Li, C. Wang, J. Zhai, High energy storage performance and fast discharging speed in dense 0.7Bi_0.5_K_0.5_TiO_3_-0.3SrTiO_3_ ceramics via a novel rolling technology. Ceram. Int. **46**(5), 6995-6998 (2020).

112. P. Zhao, B. Tang, Z. Fang, F. Si, C. Yang et al., Improved dielectric breakdown strength and energy storage properties in Er2O3 modified Sr_0.35_Bi_0.35_K_0.25_TiO_3_. Chem. Eng. J. **403**, 126290 (2021).

113. Q. Yang, M. Zhu, Q. Wei, M. Zhang, M. Zheng et al., Excellent energy storage performance of K_0.5_Bi_0.5_TiO_3_-based ferroelectric ceramics under low electric field. Chem. Eng. J. **414**, 128769 (2021).

114. L. Chen, F. Long, H. Qi, H. Liu, S. Deng et al., Outstanding Energy Storage Performance in High‐Hardness (Bi_0.5_K_0.5_)TiO_3_‐Based Lead‐Free Relaxors via Multi‐Scale Synergistic Design. Adv. Funct. Mater. **32**(9), 2110478 (2022).

115. D. Hu, Z. Pan, X. Zhang, H. Ye, Z. He et al., Greatly enhanced discharge energy density and efficiency of novel relaxation ferroelectric BNT–BKT-based ceramics. J. Mater. Chem. C **8**(2), 591-601 (2020).

116. T. Li, P. Chen, R. Si, F. Li, Y. Guo et al., High energy storage density and efficiency with excellent temperature and frequency stabilities under low operating field achieved in Ag_0.91_Sm_0.03_NbO_3_-modified Na_0.5_Bi_0.5_TiO_3_-BaTiO_3_ ceramics. J. Mater. Sci.: Mater. Electron. **31**(19), 16928-16937 (2020).

117. F. Zhang, X. Qiao, Q. Shi, X. Chao, Z. Yang et al., High energy storage density realized in Bi_0.5_Na_0.5_TiO_3_-based relaxor ferroelectric ceramics at ultralow sintering temperature. J. Eur. Ceram. Soc. **41**(1), 368-375 (2021).

118. B. Hu, H. Fan, L. Ning, Y. Wen, C. Wang, High energy storage performance of [(Bi_0.5_Na_0.5_)_0.94_Ba_0.06_]_0.97_La_0.03_Ti_1-_*_x_*(Al_0.5_Nb_0.5_)*_x_*O_3_ ceramics with enhanced dielectric breakdown strength. Ceram. Int. **44**(13), 15160-15166 (2018).

119. C. Zhu, Z. Cai, B. Luo, L. Guo, L. Li et al., High temperature lead-free BNT-based ceramics with stable energy storage and dielectric properties. J. Mater. Chem. A **8**(2), 683-692 (2020).

120. L. Zhang, X. Pu, M. Chen, S. Bai, Y. Pu, Influence of BaSnO_3_ additive on the energy storage properties of Na_0.5_Bi_0.5_TiO_3_-based relaxor ferroelectrics. J. Eur. Ceram. Soc. **38**(5), 2304-2311 (2018).

121. L. Zhang, Y. Pu, M. Chen, Influence of BaZrO_3_ additive on the energy-storage properties of 0.775Na_0.5_Bi_0.5_TiO_3_-0.225BaSnO_3_ relaxor ferroelectrics. J. Alloys Compd. **775**, 342-347 (2019).

122. Z. Liu, A. Zhang, S. Xu, J. Lu, B. Xie et al., Mediating the confliction of polarizability and breakdown electric-field strength in BNST relaxor ferroelectric for energy storage applications. J. Alloys Compd. **823**, 153772 (2020).

123. J. Wu, A. Mahajan, L. Riekehr, H. Zhang, B. Yang et al., Perovskite Sr*_x_* (Bi_1−_*_x_*Na_0.97−_*_x_*Li_0.03_)_0.5_TiO_3_ ceramics with polar nano regions for high power energy storage. Nano Energy **50**, 723-732 (2018).

124. D. Hu, Z. Pan, Z. He, F. Yang, X. Zhang et al., Significantly improved recoverable energy density and ultrafast discharge rate of Na_0.5_Bi_0.5_TiO_3_-based ceramics. Ceram. Int. **46**(10), 15364-15371 (2020).

125. L. Zhang, Y. Pu, M. Chen, Ultra-high energy storage performance under low electric fields in Na_0.5_Bi_0.5_TiO_3_-based relaxor ferroelectrics for pulse capacitor applications. Ceram. Int. **46**(1), 98-105 (2020).

126. J. Yin, Y. Zhang, X. Lv, J. Wu, Ultrahigh energy-storage potential under low electric field in bismuth sodium titanate-based perovskite ferroelectrics. J. Mater. Chem. A **6**(21), 9823-9832 (2018).

127. H. Qi, R. Zuo, Linear-like lead-free relaxor antiferroelectric (Bi_0.5_Na_0.5_)TiO_3_–NaNbO_3_ with giant energy-storage density/efficiency and super stability against temperature and frequency. J. Mater. Chem. A **7**(8), 3971-3978 (2019).

128. C. Zhang, W. Xiao, F. Zeng, D. Su, K. Du et al., Superior energy-storage performance in 0.85Bi_0.5_Na_0.5_TiO_3_–0.15NaNbO_3_ lead-free ferroelectric ceramics via composition and microstructure engineering. J. Mater. Chem. A **9**(16), 10088-10094 (2021).

129. X. Zhou, H. Qi, Z. Yan, G. Xue, H. Luo et al., Superior thermal stability of high energy density and power density in domain-engineered Bi_0.5_Na_0.5_TiO_3_–NaTaO_3_ relaxor ferroelectrics. ACS Appl. Mater. Interfaces **11**(46), 43107-43115 (2019).

130. R. Kang, Z. Wang, X. Lou, W. Liu, P. Shi et al., Energy storage performance of Bi_0.5_Na_0.5_TiO_3_-based relaxor ferroelectric ceramics with superior temperature stability under low electric fields. Chem. Eng. J. **410**, 128376 (2021).

131. Z. Jiang, Z. Yang, Y. Yuan, B. Tang, S. Zhang, High energy storage properties and dielectric temperature stability of (1-*x*)(0.8Bi_0.5_Na_0.5_TiO_3_-0.2Ba_0.3_Sr_0.7_TiO_3_)-*x*NaNbO_3_ lead-free ceramics. J. Alloys Compd. **851**, 156821 (2021).

132. Z. He, S. Shi, Z. Pan, L. Tang, J. Zhao et al., Low electric field induced high energy storage capability of the free-lead relaxor ferroelectric 0.94Bi_0.5_Na_0.5_TiO_3_-0.06BaTiO_3_-based ceramics. Ceram. Int. **47**(8), 11611-11617 (2021).

133. M.K. Bilal, R. Bashir, S.U. Asif, J. Wang, W. Hu, Enhanced energy storage properties of 0.7Bi_0.5_Na_0.5_TiO_3_-0.3SrTiO_3_ ceramic through the addition of NaNbO_3_. Ceram. Int. **47**(21), 30922-30928 (2021).

134. H. Yang, J. Tian, Y. Lin, J. Ma, Realizing ultra-high energy storage density of lead-free 0.76Bi_0.5_Na_0.5_TiO_3_-0.24SrTiO_3_-Bi(Ni_2/3_Nb_1/3_)O_3_ ceramics under low electric fields. Chem. Eng. J. **418**, 129337 (2021).

135. P. Shi, X. Zhu, X. Lou, B. Yang, X. Guo et al., Bi_0.5_Na_0.5_TiO_3_-based lead-free ceramics with superior energy storage properties at high temperatures. Comp. Part B: Eng. **215**, 108815 (2021).

136. X. Guo, P. Shi, X. Lou, Q. Liu, H. Zuo, Superior energy storage properties in (1−*x*)(0.65Bi_0.5_Na_0.5_TiO_3_-0.35Bi_0.2_Sr_0.7_TiO_3_)-*x*CaZrO_3_ ceramics with excellent temperature stability. J. Alloys Compd. **876**, 160101 (2021).

137. M. Wang, Q. Feng, Y. Wei, N. Luo, C. Yuan et al., Relaxor ferroelectric Bi_0.5_Na_0.5_TiO_3_–Sr_0.7_Nd_0.2_TiO_3_ ceramics with high energy storage density and excellent stability under a low electric field. J. Phys. Chem. Solids **157**, 110209 (2021).

138. K. Yao, C. Zhou, J. Wang, Y. Tan, Q. Li et al., Bi_0.5_Na_0.5_TiO_3_–Sr_0.85_Bi_0.1_TiO_3_ ceramics with high energy storage properties and extremely fast discharge speed via regulating relaxation temperature. Ceram. Int. **47**(8), 11294-11303 (2021).

139. C. Zhu, Z. Cai, B. Luo, X. Cheng, L. Guo et al., Multiphase engineered BNT-based ceramics with simultaneous high polarization and superior breakdown strength for energy storage applications. ACS Appl. Mater. Interfaces **13**(24), 28484-28492 (2021).

140. L. Zheng, P. Sun, P. Zheng, W. Bai, L. Li et al., Significantly tailored energy-storage performances in Bi_0.5_Na_0.5_TiO_3_–SrTiO_3_-based relaxor ferroelectric ceramics by introducing bismuth layer-structured relaxor BaBi_2_Nb_2_O_9_ for capacitor application. J. Mater. Chem. C **9**(15), 5234-5243 (2021).

141. B. Guo, Y. Yan, M. Tang, Z. Wang, Y. Li et al., Energy storage performance of Na_0.5_Bi_0.5_TiO_3_ based lead-free ferroelectric ceramics prepared via non-uniform phase structure modification and rolling process. Chem. Eng. J. **420**, 130475 (2021).

142. M. Zhang, H. Yang, D. Li, Y. Lin, Excellent energy density and power density achieved in K_0.5_Na_0.5_NbO_3_-based ceramics with high optical transparency. J. Alloys Compd. **829**, 154565 (2020).

143. M. Zhang, H. Yang, D. Li, L. Ma, Y. Lin, Giant energy storage efficiency and high recoverable energy storage density achieved in K_0.5_Na_0.5_NbO_3_-Bi(Zn_0.5_Zr_0.5_)O_3_ ceramics. J. Mater. Chem. C **8**(26), 8777-8785 (2020).

144. Z. Yang, F. Gao, H. Du, L. Jin, L. Yan et al., Grain size engineered lead-free ceramics with both large energy storage density and ultrahigh mechanical properties. Nano Energy **58**, 768-777 (2019).

145. Q. Chai, D. Yang, X. Zhao, X. Chao, Z. Yang, Lead‐free (K,Na)NbO_3_‐based ceramics with high optical transparency and large energy storage ability. J. Am. Ceram. Soc. **101**(6), 2321-2329 (2018).

146. B. Qu, H. Du, Z. Yang, Lead-free relaxor ferroelectric ceramics with high optical transparency and energy storage ability. J. Mater. Chem. C **4**(9), 1795-1803 (2016).

147. Q. Jia, Y. Li, L. Guan, H. Sun, Q. Zhang et al., Photochromic and energy storage properties in K_0.5_Na_0.5_NbO_3_-based ferroelectrics. J. Mater. Sci.: Mater. Electron. **31**(21), 19277-19292 (2020).

148. T. Shao, H. Du, H. Ma, S. Qu, J. Wang et al., Potassium–sodium niobate based lead-free ceramics: novel electrical energy storage materials. J. Mater. Chem. A **5**(2), 554-563 (2017).

149. Z. Yang, H. Du, S. Qu, Y. Hou, H. Ma et al., Significantly enhanced recoverable energy storage density in potassium–sodium niobate-based lead free ceramics. J. Mater. Chem. A **4**(36), 13778-13785 (2016).

150. B. Chen, Y. Tian, J. Lu, D. Wu, X. Qiao et al., Ultrahigh storage density achieved with (1-*x*)KNN-*x*BZN ceramics. J. Eur. Ceram. Soc. **40**(8), 2936-2944 (2020).

151. Y. Zhang, R. Zuo, Excellent energy-storage performances in La_2_O_3_ doped (Na,K)NbO_3_-based lead-free relaxor ferroelectrics. J. Eur. Ceram. Soc. **40**(15), 5466-5474 (2020).

152. M. Zhang, H. Yang, Y. Yu, Y. Lin, Energy storage performance of K_0.5_Na_0.5_NbO_3_-based ceramics modified by Bi(Zn_2/3_(Nb_0.85_Ta_0.15_)_1/3_)O_3_. Chem. Eng. J. **425**, 131465 (2021).

153. J. Xing, Y. Huang, Q. Xu, B. Wu, Q. Zhang et al., Realizing high comprehensive energy storage and ultrahigh hardness in lead-free ceramics. ACS Appl. Mater. Interfaces **13**(24), 28472-28483 (2021).

154. Z. Yang, H. Du, L. Jin, Q. Hu, S. Qu et al., A new family of sodium niobate-based dielectrics for electrical energy storage applications. J. Eur. Ceram. Soc. **39**(9), 2899-2907 (2019).

155. Y. Fan, Z. Zhou, R. Liang, X. Dong, Designing novel lead-free NaNbO_3_-based ceramic with superior comprehensive energy storage and discharge properties for dielectric capacitor applications via relaxor strategy. J. Eur. Ceram. Soc. **39**(15), 4770-4777 (2019).

156. Z. Liu, J. Lu, Y. Mao, P. Ren, H. Fan, Energy storage properties of NaNbO_3_-CaZrO_3_ ceramics with coexistence of ferroelectric and antiferroelectric phases. J. Eur. Ceram. Soc. **38**(15), 4939-4945 (2018).

157. F. Pang, X. Chen, C. Sun, J. Shi, X. Li et al., Ultrahigh energy storage characteristics of sodium niobate-based ceramics by introducing a local random field. ACS Sustainable Chem. Eng. **8**(39), 14985-14995 (2020).

158. N. Qu, H. Du, X. Hao, A new strategy to realize high comprehensive energy storage properties in lead-free bulk ceramics. J. Mater. Chem. C **7**(26), 7993-8002 (2019).

159. J. Shi, X. Chen, X. Li, J. Sun, C. Sun et al., Realizing ultrahigh recoverable energy density and superior charge–discharge performance in NaNbO_3_-based lead-free ceramics via a local random field strategy. J. Mater. Chem. C **8**(11), 3784-3794 (2020).

160. J. Shi, X. Chen, C. Sun, F. Pang, H. Chen et al., Superior thermal and frequency stability and decent fatigue endurance of high energy storage properties in NaNbO_3_-based lead-free ceramics. Ceram. Int. **46**(16), 25731-25737 (2020).

161. R. Shi, Y. Pu, W. Wang, X. Guo, J. Li et al., A novel lead-free NaNbO_3_–Bi(Zn_0.5_Ti_0.5_)O_3_ ceramics system for energy storage application with excellent stability. J. Alloys Compd. **815**, 152356 (2020).

162. L. Yang, X. Kong, Z. Cheng, S. Zhang, Enhanced energy storage performance of sodium niobate-based relaxor dielectrics by a ramp-to-spike sintering profile. ACS Appl. Mater. Interfaces **12**(29), 32834-32841 (2020).

163. Z. Yang, H. Du, L. Jin, Q. Hu, H. Wang et al., Realizing high comprehensive energy storage performance in lead-free bulk ceramics via designing an unmatched temperature range. J. Mater. Chem. A **7**(48), 27256-27266 (2019).

164. J. Ye, G. Wang, M. Zhou, N. Liu, X. Chen et al., Excellent comprehensive energy storage properties of novel lead-free NaNbO_3_-based ceramics for dielectric capacitor applications. J. Mater. Chem. C **7**(19), 5639-5645 (2019).

165. M. Zhou, R. Liang, Z. Zhou, X. Dong, Superior energy storage properties and excellent stability of novel NaNbO 3-based lead-free ceramics with A-site vacancy obtained via a Bi_2_O_3_ substitution strategy. J. Mater. Chem. A **6**(37), 17896-17904 (2018).

166. M. Zhou, R. Liang, Z. Zhou, S. Yan, X. Dong, Novel sodium niobate-based lead-free ceramics as new environment-friendly energy storage materials with high energy density, high power density, and excellent stability. ACS Sustainable Chem. Eng. **6**(10), 12755-12765 (2018).

167. H. Qi, R. Zuo, A. Xie, A. Tian, J. Fu et al., Ultrahigh energy‐storage density in NaNbO_3_‐based lead‐free relaxor antiferroelectric ceramics with nanoscale domains. Adv. Funct. Mater. **29**(35), 1903877 (2019).

168. J. Chen, H. Qi, R. Zuo, Realizing Stable Relaxor Antiferroelectric and Superior Energy Storage Properties in (Na_1-_*_x_*_/2_La*_x_*_/2_)(Nb_1-_*_x_*Ti*_x_*)O_3_ Lead-Free Ceramics through A/B-Site Complex Substitution. ACS Appl. Mater. Interfaces **12**(29), 32871-32879 (2020).

169. A. Tian, R. Zuo, H. Qi, M. Shi, Large energy-storage density in transition-metal oxide modified NaNbO_3_–Bi(Mg_0.5_Ti_0.5_)O_3_ lead-free ceramics through regulating the antiferroelectric phase structure. J. Mater. Chem. A **8**(17), 8352-8359 (2020).

170. A. Xie, H. Qi, R. Zuo, Achieving Remarkable Amplification of energy-storage density in two-step sintered NaNbO_3_–SrTiO_3_ antiferroelectric capacitors through dual adjustment of local heterogeneity and grain scale. ACS Appl. Mater. Interfaces **12**(17), 19467-19475 (2020).

171. A. Xie, R. Zuo, Z. Qiao, Z. Fu, T. Hu et al., NaNbO_3_‐(Bi_0.5_Li_0.5_)TiO_3_ Lead‐Free Relaxor Ferroelectric Capacitors with Superior Energy‐Storage Performances via Multiple Synergistic Design. Adv. Energy Mater. **11**(28), 2101378 (2021).

172. H. Chen, X. Chen, J. Shi, C. Sun, X. Dong et al., Achieving ultrahigh energy storage density in NaNbO_3_–Bi(Ni_0.5_Zr_0.5_)O_3_ solid solution by enhancing the breakdown electric field. Ceram. Int. **46**(18), 28407-28413 (2020).

173. X. Dong, X. Li, X. Chen, H. Chen, C. Sun et al., High energy storage density and power density achieved simultaneously in NaNbO_3_-based lead-free ceramics via antiferroelectricity enhancement. J. Materiomics **7**(3), 629-639 (2021).

174. X. Dong, X. Li, X. Chen, H. Chen, C. Sun et al., High energy storage and ultrafast discharge in NaNbO_3_-based lead-free dielectric capacitors via a relaxor strategy. Ceram. Int. **47**(3), 3079-3088 (2021).

175. J. Jiang, X. Meng, L. Li, J. Zhang, S. Guo et al., Enhanced energy storage properties of lead-free NaNbO_3_-based ceramics via A/B-site substitution. Chem. Eng. J. **422**, 130130 (2021).

176. C. Sun, X. Chen, J. Shi, F. Pang, X. Dong et al., Simultaneously with large energy density and high efficiency achieved in NaNbO_3_-based relaxor ferroelectric ceramics. J. Eur. Ceram. Soc. **41**(3), 1891-1903 (2021).

177. H. Chen, J. Shi, X. Dong, F. Pang, H. Zhang et al., Enhanced thermal and frequency stability and decent fatigue endurance in lead-free NaNbO_3_-based ceramics with high energy storage density and efficiency. J. Materiomics **8**(2), 489-497 (2022).

178. F. Yan, H. Yang, Y. Lin, T. Wang, Dielectric and ferroelectric properties of SrTiO_3_–Bi_0.5_Na_0.5_TiO_3_–BaAl_0.5_Nb_0.5_O_3_ lead-free ceramics for high-energy-storage applications. Inorg. Chem. **56**(21), 13510-13516 (2017).

179. W. Wang, Y. Pu, X. Guo, R. Shi, Y. Shi et al., Enhanced energy storage density and high efficiency of lead-free Ca_1-_*_x_*Sr*_x_*Ti_1-_*_y_*Zr*_y_*O_3_ linear dielectric ceramics. J. Eur. Ceram. Soc. **39**(16), 5236-5242 (2019).

180. L. Zhang, Z. Wang, Y. Li, P. Chen, J. Cai et al., Enhanced energy storage performance in Sn doped Sr_0.6_(Na_0.5_Bi_0.5_)_0.4_TiO_3_ lead-free relaxor ferroelectric ceramics. J. Eur. Ceram. Soc. **39**(10), 3057-3063 (2019).

181. W. Pan, M. Cao, A. Jan, H. Hao, Z. Yao et al., High breakdown strength and energy storage performance in (Nb,Zn) modified SrTiO_3_ ceramics via synergy manipulation. J. Mater. Chem. C **8**(6), 2019-2027 (2020).

182. C. Cui, Y. Pu, R. Shi, High-energy storage performance in lead-free (0.8-*x*)SrTiO_3_-0.2Na_0.5_Bi_0.5_TiO_3_-*x*BaTiO_3_ relaxor ferroelectric ceramics. J. Alloys Compd. **740**, 1180-1187 (2018).

183. H. Yang, F. Yan, Y. Lin, T. Wang, Improvement of dielectric and energy storage properties in SrTiO_3_-based lead-free ceramics. J. Alloys Compd. **728**, 780-787 (2017).

184. C. Cui, Y. Pu, Z. Gao, J. Wan, Y. Guo et al., Structure, dielectric and relaxor properties in lead-free ST-NBT ceramics for high energy storage applications. J. Alloys Compd. **711**, 319-326 (2017).

185. X. Kong, L. Yang, Z. Cheng, S. Zhang, Ultrahigh energy storage properties in (Sr_0.7_Bi_0.2_)TiO_3_-Bi(Mg_0.5_Zr_0.5_)O_3_ lead-free ceramics and potential for high-temperature capacitors. Materials **13**(1), 180 (2020).

186. C. Cui, Y. Pu, Improvement of energy storage density with trace amounts of ZrO_2_ additives fabricated by wet-chemical method. J. Alloys Compd. **747**, 495-504 (2018).

187. H. Yang, F. Yan, Y. Lin, T. Wang, Enhanced recoverable energy storage density and high efficiency of SrTiO_3_-based lead-free ceramics. Appl. Phys. Lett. **111**(25), 253903 (2017).

188. H. Yang, F. Yan, Y. Lin, T. Wang, Novel strontium titanate-based lead-free ceramics for high-energy storage applications. ACS Sustainable Chem. Eng. **5**(11), 10215-10222 (2017).

189. X. Guo, Y. Pu, W. Wang, J. Ji, J. Li et al., Ultrahigh energy storage performance and fast charge-discharge capability in Dy-modified SrTiO_3_ linear ceramics with high optical transmissivity by defect and interface engineering. Ceram. Int. **46**(13), 21719-21727 (2020).

190. T. Cui, J. Zhang, J. Guo, X. Li, S. Guo et al., Outstanding comprehensive energy storage performance in lead-free BiFeO_3_-based relaxor ferroelectric ceramics by multiple optimization design. Acta Mater. **240**, 118286 (2022).

191. T. Cui, J. Zhang, J. Guo, X. Li, S. Guo et al., Simultaneous achievement of ultrahigh energy storage density and high efficiency in BiFeO_3_-based relaxor ferroelectric ceramics via a highly disordered multicomponent design. J. Mater. Chem. A **10**(27), 14316-14325 (2022).

192. K. Han, N. Luo, Y. Jing, X. Wang, B. Peng et al., Structure and energy storage performance of Ba-modified AgNbO_3_ lead-free antiferroelectric ceramics. Ceram. Int. **45**(5), 5559-5565 (2019).

193. D. Wang, Z. Fan, D. Zhou, A. Khesro, S. Murakami et al., Bismuth ferrite-based lead-free ceramics and multilayers with high recoverable energy density. J. Mater. Chem. A **6**(9), 4133-4144 (2018).

194. A. Xie, J. Fu, R. Zuo, X. Jiang, T. Li et al., Supercritical relaxor nanograined ferroelectrics for ultrahigh-energy-storage capacitors. Adv. Mater. **34**, 2204356 (2022).

195. H. Tan, Z. Yan, S.-G. Chen, C. Samart, N. Takesue et al., SPS prepared NN-24SBT lead-free relaxor-antiferroelectric ceramics with ultrahigh energy-storage density and efficiency. Scripta Mater. **210**, 114428 (2022).
